# Structure of biomimetic casein micelles: Critical tests of the hydrophobic colloid and multivalent-binding models using recombinant deuterated and phosphorylated β-casein

**DOI:** 10.1016/j.yjsbx.2024.100096

**Published:** 2024-01-22

**Authors:** Jared K. Raynes, Jitendra Mata, Karyn L. Wilde, John A. Carver, Sharon M. Kelly, Carl Holt

**Affiliations:** a CSIRO Agriculture & Food, 671 Sneydes Road, Werribee, VIC 3031, Australia; b All G Foods, Waterloo, NSW 2006, Australia; c Australian Centre for Neutron Scattering, Australian Nuclear Science and Technology Organisation, Lucas Heights, NSW 2234, Australia; d School of Chemistry, University of New South Wales, Sydney 2052, Australia; e National Deuteration Facility, Australian Nuclear Science and Technology Organisation, Lucas Heights, NSW 2234, Australia; f Research School of Chemistry, The Australian National University, Acton, ACT 2601, Australia; g School of Molecular Biosciences, University of Glasgow, Glasgow G12 8QQ, United Kingdom

**Keywords:** Casein micelle, Mineral chaperone, Calcium phosphate, Small-angle scattering, Intrinsically disordered protein

## Abstract

Milk contains high concentrations of amyloidogenic casein proteins and is supersaturated with respect to crystalline calcium phosphates such as apatite. Nevertheless, the mammary gland normally remains unmineralized and free of amyloid. Unlike κ-casein, β- and α_S_-caseins are highly effective mineral chaperones that prevent ectopic and pathological calcification of the mammary gland. Milk invariably contains a mixture of two to five different caseins that act on each other as molecular chaperones. Instead of forming amyloid fibrils, several thousand caseins and hundreds of nanoclusters of amorphous calcium phosphate combine to form fuzzy complexes called casein micelles. To understand the biological functions of the casein micelle its structure needs to be understood better than at present. The location in micelles of the highly amyloidogenic κ-casein is disputed. In traditional hydrophobic colloid models, it, alone, forms a stabilizing surface coat that also determines the average size of the micelles. In the recent multivalent-binding model, κ-casein is present throughout the micelle, in intimate contact with the other caseins. To discriminate between these models, a range of biomimetic micelles was prepared using a fixed concentration of the mineral chaperone β-casein and nanoclusters of calcium phosphate, with variable concentrations of κ-casein. A biomimetic micelle was also prepared using a highly deuterated and *in vivo* phosphorylated recombinant β-casein with calcium phosphate and unlabelled κ-casein. Neutron and X-ray scattering experiments revealed that κ-casein is distributed throughout the micelle, in quantitative agreement with the multivalent-binding model but contrary to the hydrophobic colloid models.

## Highlights

• Recombinant, deuterated, *in vivo* phosphorylated β-casein was produced.
• Biomimetic casein micelles were produced and characterised.
• κ-Casein was shown to be distributed throughout the casein micelle.
• Findings agree with the multivalent-binding model, not the hydrophobic colloid models.
• Casein micelle acts a mineral and protein chaperone in the mammary gland.

## Graphical abstract

**Figure:**
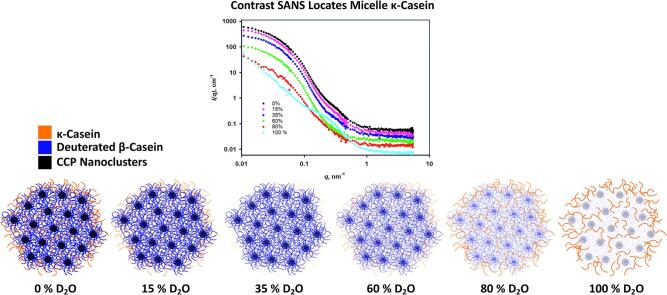

## Introduction

The role of phosphoproteins in the control of biocalcification is widely recognised (Alvares, 2014, Coelfen, 2010, Deshpande et al., 2011, Edén, 2021, Evans, 2013, Gal et al., 2015, Jahnen-Dechent and Smith, 2020, Jahnen-Dechent et al., 2020, Jin et al., 2018, Kahil et al., 2021, Kawasaki et al., 2009, Nudelman et al., 2012, Prasad et al., 2010, Qin et al., 2022, Rao et al., 2018, Weiner and Addadi, 2011). An example of fine spatial control of tissue mineralization by phosphoproteins occurs at the boundary formed by the periodontal ligament which remains unmineralized even though it lies between two mineralized tissues, cementum and alveolar bone (Foster et al., 2018, Zuo et al., 2019). Another example is the stabilization by phosphoproteins of biofluids such as blood, extracellular fluid, saliva and milk which are supersaturated with respect to the apatite phases of bones and teeth (Holt et al., 2014), but they do not normally cause the soft tissues with which they are in contact to become mineralized (Holt, 2013). An explanation can be formulated using the chemistry of calcium carbonate or calcium phosphate (CaP). Specifically, at physiological pH these salts can precipitate from a supersaturated solution by a non-classical mechanism through the growth of ion pairs into pre-nucleation clusters (Garcia et al., 2019, Gebauer et al., 2018, Weber et al., 2020) leading to an initial amorphous phase (Gebauer et al., 2018). The rate and extent of formation of the initial highly hydrated amorphous phase and its maturation into more stable crystalline phases can be influenced by the concentration and degree of phosphorylation of phosphoproteins to an extent that would not be possible for classical nucleation mechanisms (de Bruyn et al., 2013, George and Veis, 2008, Jiang et al., 2012, Rao et al., 2018, Yu and Wei, 2021). Mineral chaperones that inhibit or promote mineralization (Hunter et al., 2014) in a tissue-specific manner, are usually phosphorylated intrinsically disordered proteins (IDPs) (Boskey and Villarreal-Ramirez, 2016, Holt et al., 2009, Hunter et al., 2010, Kalmar et al., 2012). Examples include fetuin A (Jahnen-Dechent et al., 2020, Jahnen-Dechent and Smith, 2020) in blood, the caseins of milk and many other members of the paralogous group to which caseins belong, of secreted calcium- or CaP-binding phosphoproteins (SCPPs) (Kawasaki and Weiss, 2003, Kawasaki and Weiss, 2008). SCPPS such as osteopontin are very widely distributed in species, tissues and biofluids.

### Mineral chaperone of blood

The CaP complexes with Fetuin A, called calciprotein particles of types I and II, are responsible for maintaining blood mineral saturation, controlling ectopic calcification of the vasculature, and are implicated in the maintenance of bone mineralization (Akiyama et al., 2020, Jahnen-Dechent and Smith, 2020, Jahnen-Dechent et al., 2020). They are initially spherical, amorphous and soft (Koppert et al., 2018) and have been likened to an extracellular membrane-less organelle (Jahnen-Dechent and Smith, 2020, Jahnen-Dechent et al., 2020), but they are metastable under physiological conditions and mature into more oblate structures containing more crystalline and less soluble forms of CaP. Glomerular filtration of calciprotein particles removes them from the circulation so that there are typically about 10^9^-10^10^ particles per litre of blood (Akiyama et al., 2020, Jahnen-Dechent et al., 2020, Jahnen-Dechent and Smith, 2020).

### Mineral and protein chaperones of milk

In contrast to the blood calciprotein particles, the CaP complexes with Ca-sensitive caseins are extremely long-lived and resistant to conversion into less soluble and more crystalline forms (Lenton et al., 2020). The mineral chaperones in milk are the SCPPs known as caseins (Kawasaki et al., 2011). The naturally occurring complexes of caseins and CaP are the casein micelles (which have no structural similarity to detergent micelles). They form a polydisperse assembly of colloidal particles, typically containing several hundred CaP-nanoclusters together with about 10,000 caseins per micelle in what has been described as either a fuzzy, non-stoichiometric, complex of IDPs (Holt, 2021, Holt and Carver, 2022) or, nearly equivalently, as an extracellular condensed phase (Horvath et al., 2022). All natural casein micelles contain some κ-casein, but in other respects their composition is highly variable and each of the other caseins may be absent or undetectable in certain species or individuals.

An important post-translational modification of caseins is phosphorylation to different degrees of (usually) serine residues (Miranda et al., 2020), mostly by the Golgi kinase Fam20C (Tagliabracci et al., 2015). CaP nanoclusters do not exist in the micelle as independent entities but are sequestered by a shell of bound caseins to form CaP nanocluster complexes (Lenton et al., 2020). The binding is predominantly through short,highly phosphorylated, serine-rich sequences denoted CaP-SLiMs (De Sa Peixoto et al., 2017, Holt, 2021, Holt and Carver, 2022, Ono et al., 1990). For example, the sequence 14-E-pS-L-pS-pS-pS-E-E-21, where pS is phosphoserine, is the CaP-SLiM in cow β-casein (numbering is after removal of the N-terminal signal sequence of 15 amino acids). The cow β β-, α_S1_- and α_S2_-contain one or more similar CaP-SLiMs and are called Ca-sensitive caseins because they are precipitated by low mM concentrations of calcium salts under physiological conditions. By contrast, cow κ-casein usually has only one phosphorylated residue and it is not precipitated by low mM concentrations of calcium salts. As a result, κ-casein is called a Ca-insensitive casein. In cow milk, about 70 % of the Ca-sensitive caseins are bound to the CaP nanoclusters (Aoki et al., 1991, Holt and Carver, 2022). The remaining free Ca-sensitive caseins are mostly in the micelles but can readily exchange with the surrounding milk serum.

Caseins can also act as non-ATP-dependent, holdase-type molecular chaperones to limit globular protein unfolding and aggregation under stress conditions (Bhattacharyya and Das, 1999, Morgan et al., 2005). All four of the cow caseins can form amyloid fibrils (Bahraminejad et al., 2022, Farrell et al., 2003, Thorn et al., 2005, Treweek et al., 2011) but because of their action as molecular chaperones a mixture of two or more different caseins can instead form an amorphous oligomeric structure. Indeed, it has been suggested that the native casein micelle, invariably a mixture of two – five caseins, is the means by which the mammary tissue remains free of amyloid structures even though milk contains mM concentrations of amyloidogenic caseins (Bahraminejad et al., 2022),

### Structure of the native casein micelle

As a result of scattering and other non-perturbing structural methods (de Kruif, 2014, Marchin et al., 2007, Morris et al., 2000, Nogueira et al., 2021), a widely accepted medium-resolution structural model of the native casein micelle has been obtained. Casein micelles from cow milk are roughly spherical with a number average radius of about 75 nm. A histogram of the size distribution, determined by nanoparticle tracking analysis, is unimodal with sizes ranging from 10 to several hundred nm (Holt, 2021, Saveyn et al., 2010). The weight average molecular mass is typically about 2 − 5x10^8^ Da (Dewan et al., 1974, Hansen et al., 1996, Holt et al., 1978, Morris et al., 2000). The micelle is highly hydrated by bound and entrained water molecules, giving it a voluminosity of about 4.1 mL g^−1^ at room temperature (Nobel et al., 2012). In other words, 70–80 % of the volume of a casein micelle is water. Notwithstanding the high hydration, a solute volume fraction of 0.2 is sufficiently high for the micelle to have viscoelastic properties (Bouchoux et al., 2009, Uricanu et al., 2004). Cryo-electron microscopy of raw milk micelles shows that the CaP nanocluster complexes are distributed more-or-less evenly through the core of the micelle (de Kruif et al., 2012, Hettiarachchi et al., 2020, Kamigaki et al., 2018, Marchin et al., 2007) but they are not found in the coat (Bouchoux et al., 2015, Shukla et al., 2009). The casein micelle is a dynamic structure: individual caseins are conformationally mobile and can exchange between the micelle and milk serum, particularly those that are not bound strongly to the CaP nanoclusters (Nogueira et al., 2021). The internal structure and size distribution also respond to changes of pH, temperature and pressure, or additions of salts, polyphenols, and ethanol ((Bouchoux et al., 2010, Day et al., 2017, Hansen et al., 1996, Khanji et al., 2015, Knudsen and Skibsted, 2010, Saveyn et al., 2010, Takagi et al., 2022, Yang et al., 2021, Yang et al., 2018). According to the hydrophobic colloid model of casein micelle structure, a monolayer of κ-casein forms a coat around a core of the supposedly more hydrophobic caseins (Holt and Horne, 1996, Talbot and Waugh, 1967) and thereby determines the average size of casein micelles. In the recent multivalent-binding model, all free caseins, including κ-casein, are present in the coat and core (Holt, 2021).

We used small angle X-ray (SAXS) and small angle neutron scattering (SANS) with contrast variation to examine how casein micelle size depends on the concentration of κ-casein or CaP nanocluster concentration in highly controlled experiments where other factors affecting micelle size are carefully controlled. In addition, the contrast-variation SANS data provided specific information on the location of κ-casein within the casein micelle.

## Materials and methods

### Cow β- and κ-caseins

κ-Casein was prepared from the skim-milk of an individual Ayrshire cow by the method of (Zittle and Custer, 1963) followed by chromatography in 6 M urea on a Sephadex SPC 25 column (Annan and Manson, 1969). β-Casein A^2^ was sourced from an individual cow from the research herd maintained at the Agriculture Victoria Ellinbank Smartfarm at Ellinbank, Victoria, Australia and purified as previously described (Raynes et al., 2015).

### Recombinant β-casein

r-β-casein 4-P (containing four phosphoserines) was obtained by adapting the procedure of (Clegg and Holt, 2009) which used a dual plasmid transformation of *E. coli to* co-express a β-casein analogue with the catalytic α subunit of casein kinase II. The CaP-SLiM near the N-terminus (residues 1–21) had additional flanking Asp residues to promote phosphorylation by casein kinase II.

## Deuteration of recombinant β-casein

Deuterated r-β-casein (r-D-β-casein) was prepared using a high cell density variation of the method of Duff et al., 2015 [1 1 5], starting from a glycerol stock of co-transformed *E. coli* cells. Host cells were adapted in three culturing steps using ModC1 minimal medium with increasing D_2_O concentration, while increasing culture volume from 10 to 100 mL. At an OD_600_ of 0.8 (10 mm path length) the 100 mL culture in 99 % (v/v) D_2_O minimal medium was inoculated into a further 900 mL of fresh 99 % (v/v) D_2_O ModC1 medium with 40 gL^-1^ glycerol in a 2 L bioreactor. The *E. coli* cells were grown until OD_600_ reached 15. Expression was induced by adding isopropyl-β-D-thiogalactoside to a final concentration of 1 mM. After 22 h at 20 °C the deuterated cell suspension was centrifuged at 8000 × *g* for 30 min and the pelleted cells stored frozen at −80 °C.

Purification was by the method of (Clegg and Holt, 2009) except that the final stage used anion exchange chromatography rather than preparative scale RP-HPLC. The filtered cell lysate was loaded onto a GE Healthcare HiTrap HP Q 5 mL column (ÄKTA™ PURE 25 GE Healthcare Bio-Sciences, Switzerland) pre-equilibrated with buffer A (6 M Urea, 20 mM Tris pH 8.0) at a flow rate of 5 mL/min. Elution was monitored at 280 nm. The bound protein was washed with 5 column volumes of buffer A before a linear gradient of buffer B (6 M Urea, 20 mM Tris, 1 M NaCl pH 8.0) was applied over 50 column volumes until 40 % v/v buffer B. Fractions were collected to identify the purified protein by SDS-PAGE which eluted at 0.33 M NaCl. Both r-H-β-casein and r-D-β-casein eluted at the same NaCl concentration suggesting that they carried a similar number of phosphorylated residues. This conclusion is strongly supported by the previous characterisation of the phosphoforms of the recombinant N-terminal phosphopeptide of β-casein (Clegg and Holt, 2009) co-expressed with casein kinase II.

### Determination of protein masses using mass spectrometry

Electrospray ionisation quadruple time-of-flight mass spectrometry was carried out on an UltiMate 3000 HPLC system (Thermo Fisher Scientific) coupled to a MaXis II Q-TOF mass spectrometer (Bruker Daltonics, MA USA) to identify the degree of phosphorylation. In short, the purified samples (1 gL^-1^) in H_2_O were diluted with 0.1 % v/v formic acid and loaded onto a 50 x 4.6 mm, 5 μM particle size, 300 Å pore size Agilent PLRP-S column, pre-equilibrated with 0.1 % v/v formic acid. The protein was eluted from the column at a flow rate of 250 μL min^−1^ by applying a linear gradient from 0 to 80 % mobile phase B (mobile phase A: 0.1 % (v/v) formic acid; mobile phase B: 90 % (v/v) acetonitrile/0.1 % (v/v) formic acid) and ionised using an Apollo II electron spray ion source (Bruker Daltonics) with nebulizer pressure set at 1.8 Bar and dry gas maintained at 220 °C at a flow rate of 8 L/min. High-resolution LC-MS data were analysed using the intact mass parsimonious charge-state deconvolution algorithm (Bern et al., 2018).

### Degree of phosphorylation and deuteration of recombinant β-casein

There are four Ser residues in consensus casein kinase-II sites in the r-β-casein analogue with a potential fifth Ser residue in a non-consensus site (Clegg and Holt, 2009). Deconvolution of the whole protein mass spectrum of r-H-β-casein confirmed that the main species was the 4-P phosphoform with much smaller peaks corresponding to 2-, 3- and 5-P phosphoforms and some other, minor, unidentified, species. Thus, the r-β-casein 4-P protein has a CaP-SLIM closely comparable to the single CaP-SLiM in native cow β-casein (residues 14–21).

The deconvolved mass spectrum of the r-D-β-casein (i. e. the highly deuterated form) has a broad peak with its maximum at 25185.8 Da. Mass spectrometry and purification were carried out in H_2_O solutions allowing exchangeable protons to equilibrate with the solvent. There are 302–336 labile protons out of a total of 1669 hydrogen atoms in r-β-casein, depending on pH, which will exchange with the solvent. In r-β-casein 4-P, these numbers are 310–340 out of a total of 1677. From the peak mass of the deconvolved mass spectrum, the degree of deuteration of the r-D-β-casein 4P was calculated to be between 0.89 and 0.91.

### Physico-chemical properties of the recombinant β-casein

Like the native β-casein, the recombinant β-casein showed an endothermic transition at low temperature which has been ascribed to a small conformational change associated with the temperature-dependent-self association of the protein (Supplementary Materials Section 3). The transition was more endothermic and at a lower temperature than for native β-casein. Previously, it was observed that deletion of the C-terminal sequence of β β-casein reduced self-association (Qi et al., 2004) but our findings strongly suggest that the N-terminal region is also involved and therefore self-association of β-casein is likely to involve multivalent interactions. The far-UV circular dichroism spectrum of recombinant β-casein (Supplementary Materials Section 3) was similar to that of native β-casein and other IDPs in containing a high proportion of poly-L-proline II secondary structure (Syme et al., 2002). The ability of the N-terminal sequence of the recombinant casein to sequester CaP and form CaP nanocluster complexes was demonstrated previously (Clegg and Holt, 2009) to be very similar to the behaviour of the N-terminal tryptic peptide of native β-casein (Holt et al., 1998).

### Preparation of the biomimetic casein micelles from β- and κ-caseins and CaP nanoclusters

In the range of samples, designated B8Kx, the β-casein concentration was held constant at 8 gL^-1^ and the κ-casein concentration, x, was in the range 2⩽x⩽20 gL^-1^. Preliminary work, described in Supplementary Materials Section 3, and previous work (Clegg and Holt, 2009) established that the r-β-casein was phosphorylated at a similar level to native β-casein, that it is an IDP with a predominance of the poly-L-proline secondary structure and that it associates endothermically, like native cow β-casein. Further preliminary work, described in Supplementary Materials Section 4, established that the number of casein components required to form a biomimetic stable casein micelle could be reduced to two, one of these being a Ca-sensitive casein with a single CaP-SLiM (β-casein) and the second the Ca-insensitive κ-casein. Like native casein micelles, the biomimetic B8Kx micelles decreased in average size with increase in x and were further comparable to native micelles in being spherical particles with a similar scale of substructure. Likewise, their substructure was sensitive to pH and, at neutral pH, was enhanced by the addition of 5 – 10 % ethanol. For these reasons we consider the B8Kx particles, prepared by the urea/urease method, to mimic the properties of native casein micelles. The great advantage of using the biomimetic casein micelles is that they have a defined composition and can be used in critical tests of casein micelle structure.

The most important parameters affecting casein micelle size are the concentration of CaP-nanoclusters, which determines the fraction of bound β-casein (α) to the CaP-nanoclusters, the free calcium ion concentration, pH and ionic strength. These were all controlled within narrow limits so that the only important variable affecting micelle size within a given series was the concentration of κ-casein.

Two series of samples were prepared and used to measure the dependence of size on the κ-casein concentration, one with α close to unity and one with α close to 0.5. SAXS and SANS were used to measure the relationship between radius of gyration and the κ-casein concentration. A further biomimetic casein micelle was prepared with α nominally 0.5, denoted r-D-B8K2. It contained 8 gL^-1^ of a fully phosphorylated and deuterated recombinant analogue of β-casein (Clegg and Holt, 2009) and 2 gL^-1^ of unlabelled κ-casein. Because of the increased molar mass of the r-D-β-casein, its molar concentration in r-D-B8K2 was smaller than in B8K2 and as a result a higher proportion of the β-casein analogue was bound directly to the CaP nanoclusters (α ∼ 0.624 compared to 0.565 for B8K2). This sample was used in a SANS experiment with contrast variation.

The salts used to make the CaP nanoclusters were a selection of those in milk. Samples with α ∼ 0.5 had a pH of 6.75 ± 0.05 and contained 7.1 mM total Ca, 6.5 mM total inorganic orthophosphate (P_i_). Approximately 36 mM NaCl, was added to give a constant ionic strength of 0.05 in the continuous phase.

The composition of a dilution buffer matching that of the continuous phase and the partition of salts and between the micelles and continuous phase and the partition of β-casein between bound and free states were calculated using the method of (Bijl et al., 2019, Holt, 2021). It was previously established (Little and Holt, 2004) that in citrate-free media the empirical chemical formula of the CaP in CaP nanocluster complexes with short casein phosphopeptides is Ca(HPO_4_)_0.4_(PO_4_)_0.4_, which means it is an acidic form of amorphous CaP.

To allow samples to be diluted to a suitable concentration for scattering measurements, without disrupting the micelles, dilution buffers were prepared which matched as closely as possible the composition of the continuous phase of the samples. Their composition was 36 mM NaCl, 1.5 mM NaN_3_, 4.4 mM inorganic orthophosphate (56.8 % v:v NaH_2_PO_4_^-^, 43.2 % v:v Na_2_HPO_4_^-^) and 2.93 mM CaCl_2_. The dilution buffers are supersaturated with respect to crystalline phases of CaP, so they were stabilised against precipitation by adding a mixture of short casein phosphopeptides (Lacprodan® DI-2090 from Arla Foods) at a concentration of 1 g/L. Dilution buffers were stirred overnight at 25 °C and examined to ensure no precipitation had occurred.

The partition of salts between the casein micelles and continuous phase was determined experimentally to provide a check on the accuracy of the salt partition calculations and for use in the modelling work. After equilibration of the CO_2_ with air for 24–72 h, serum concentrations were determined by ultrafiltration using VivaSpin divided centrifuge tubes with a membrane mass cut-off of 10,000 Da, as previously described (Little and Holt, 2004). The sample and serum compositions are summarised in Table 1.

**Table 1:** Calculated and experimental properties of B8Kx micelles with α ∼ 0.51.

	**B8K2**	**B8K3**	**B8K5**	**B8K10**	**r-D-B8K2**
Calculated diffusible Ca, mM	3.39	3.37	3.33	3.26	3.41
Experimental diffusible Ca, mM	2.93	2.90	3.11	3.05	–
Calculated diffusible P_i_, mM	4.90	4.92	4.96	5.06	4.82
Experimental diffusible P_i_, mM	4.40	4.41	4.38	4.37	–
Calculated free Ca^2+^, mM	2.55	2.54	2.50	2.44	2.57
Salt partition parameter,$\varphi_{\mathrm{core}}$	4.00	5.00	7.10	13.5	3.79
Fraction of bound β-casein, α	0.57	0.56	0.55	0.51	0.62
Self-association affinity,$K_{\mathrm{core}}x_{1}$	0.84	0.725	0.50	0.43	0.992
Free/bound caseins	1.30	1.59	2.21	3.91	1.11
β-casein/ κ-casein in the coat	1.31	0.98	0.60	0.33	1.19
Charge per core protein	−5.53	−5.39	−5.18	−4.85	−8.17
Charge per coat protein	−6.82	−6.31	−5.70	−5.02	−8.54
Weight average core radius, nm	23.7	19.4	15.3	14.5	70.3
Weight average mass, 10^-6^ Da	25.4	15.5	9.0	10.2	6.4
Number average mass, 10^-6^ Da	14.2	9.2	6.1	8.0	3.2

1 All model calculations used$K_{\mathrm{core}}x_{0}=1$.

The range of samples with α≃1.0 contained 9 mM total Ca, 5 mM total Mg, 10.0 mM total orthophosphate and had an ionic strength of 0.07. The calculated free calcium and magnesium ion concentrations were 2.0 and 0.9 mM, respectively. No experimental salt partition measurements were made on these samples or on the r-D-B8K2.

### Contrast variation samples

The r-D-B8K2 biomimetic micelles were chosen for contrast variation SANS studies because the SAXS and SANS experiments with B8K2 prepared with the cow proteins indicated they would be sufficiently large to be compared to cow casein micelles but small enough for the Guinier region to be readily accessible. Stock samples and dilution buffers were prepared in the same way as those for SAXS studies but using either H_2_O or D_2_O and adjusting each to a pH meter reading of 6.7. Samples and dilution buffers were prepared by mixing the stocks in different proportions. To match the solvent and diffusible salt concentrations of stock sample and dilution buffer, 1 mL of each stock sample was placed in a Spectra/Por® Float-A-Lyzer® G2 (3.5 kDa cut-off) and dialysed against 50 mL of the corresponding dilution buffer for 5 h. A range of neutron scattering contrasts was created by mixing stock dialysed sample solutions to give 0, 20, 40, 60, 80 and 100 % v:v D_2_O. Corresponding dilution buffers were prepared by mixing the dialysed stock dilution buffers in the same proportions.

### Small-angle X-ray scattering

The SAXS measurements were undertaken at the SAXS/WAXS beamline of the Australian Synchrotron (Clayton, Melbourne, Australia). The beamline was equipped with a Pilatus 1 M detector (170 mm x 170 mm, effective 231pixel size of 172 x 172 μm). Each sample was measured at sample-to-detector lengths of 7.106 m and 0.721 m with photon energies of 8.2 keV (1.512 Å) or 18.1 keV (0.685 Å), respectively. Merging the data from both camera lengths provided a *q* range of 1.3∙10^-3^ to 1.93 Å-^1^. The samples were drawn into a 1.5 mm glass capillary, allowing continuous flow through the X-ray beam during measurements. The data were obtained by averaging at least 10 exposures of 2 s at 20 °C. The capillary was rinsed between samples with water, followed by 8 M guanidine hydrochloride and then with water before being air-dried. The SAXS intensities were normalized to an absolute scale and the corresponding solvent background subtracted using ScatterBrain (V 2.71) (Australian Synchrotron, Clayton, Australia).

### Small-angle neutron scattering

SANS experiments were undertaken on the Quokka SANS 40 m instrument at the Australian Nuclear Science & Technology Organisation (ANSTO), Lucas Heights, NSW, Australia. A detailed description of the instrument is given in (Wood et al., 2018). Three camera lengths were used: 20 m with lens configuration (8.1 Å incident neutron), 12 m and 1.3 m (5 Å incident neutron), giving a *q* range of 0.00088 – 0.54883 Å^−1^. A temperature-controlled 20-cell holder was used at 25 °C with 1 mm or 2 mm optical path length cylindrical quartz cells. Sample exposure times and other SANS parameters are given in Supplementary Material, Section 2. Buffer background exposure times were the same as for the samples in H_2_O.

### Scattering length densities

For neutron and X-ray scattering, the scattering length densities of the solvent, caseins and the nanocluster CaP were obtained from the scattering length density calculator on the NIST website (https://www.ncaseinr.nist.gov/resources/activation/). The results of the scattering length density calculations for the individual components of a micelle can be found in Supplementary Material Section 1.

### Determination of the radius of gyration of casein micelles from scattering data

Casein micelles are physically and chemically heterogeneous because the coat and core differ in average solute scattering length density and hydration. As a result, the relative contribution of the core and coat to the scattering by each *j-*mer varies with *j*. We assume that the composition and hydration of the coat and core are the same for all *j*-mers. The multivalent-binding model provides expressions for the solute (casein plus CaP) composition of the coat and core (Holt, 2021, Holt and Carver, 2022) allowing their average scattering length densities, ${\overset{¯}{\rho }}_{\mathrm{coat}}$ and ${\overset{¯}{\rho }}_{\mathrm{core}}$, respectively, to be calculated. Let the solute volume fractions in the coat and core be $\phi_{\mathrm{coat}}$ and $\phi_{\mathrm{core}}$, respectively, so that the excess scattering length densities are $\Delta {\overset{¯}{\rho }}_{\mathrm{coat}}=\phi_{\mathrm{coat}}\left({\overset{¯}{\rho }}_{\mathrm{coat}}-\rho_{s}\right)$ and $\Delta {\overset{¯}{\rho }}_{\mathrm{core}}=\phi_{\mathrm{core}}\left({\overset{¯}{\rho }}_{\mathrm{core}}-\rho_{s}\right)$. The square of the radius of gyration of a *j*-mer is

(1) $$R_{g}^{2}\left(j\right)=\frac{3}{5}\frac{\phi_{\mathrm{coat}}\left({\overset{¯}{\rho }}_{\mathrm{coat}}-\rho_{s}\right)R_{\mathrm{coat}}^{5}\left(j\right)+\left(\phi_{\mathrm{core}}{\overset{¯}{\rho }}_{\mathrm{core}}-\phi_{\mathrm{coat}}{\overset{¯}{\rho }}_{\mathrm{coat}}\right)R_{\mathrm{core}}^{5}\left(j\right)}{\phi_{\mathrm{coat}}\left({\overset{¯}{\rho }}_{\mathrm{coat}}-\rho_{s}\right)R_{\mathrm{coat}}^{3}\left(j\right)+\left(\phi_{\mathrm{core}}{\overset{¯}{\rho }}_{\mathrm{core}}-\phi_{\mathrm{coat}}{\overset{¯}{\rho }}_{\mathrm{coat}}\right)R_{\mathrm{core}}^{3}\left(j\right)}$$

Which simplifies to the usual expression for a core–shell particle when $\phi_{\mathrm{coat}}=\phi_{\mathrm{core}}$ (Pedersen, 1997).

For a polydisperse distribution, the intensity-weighted average of the square of the radius of gyration, $\overset{¯}{R_{g}^{2}}$, is obtained from a small-angle scattering experiment.

(2) $$\overset{¯}{R_{g}^{2}}=\frac{\sum_{j=1}^{j_{\max}}N\left(j\right)\Delta {\overset{¯}{\rho }}^{2}\left(j\right)M^{2}\left(j\right)R_{g}^{2}\left(j\right)}{\sum_{j=1}^{j_{\max}}N\left(j\right)\Delta {\overset{¯}{\rho }}^{2}\left(j\right)M^{2}\left(j\right)}$$

Where $\Delta \overset{¯}{\rho }\left(j\right)=\overset{¯}{\rho }\left(j\right)-\rho_{s}$ is the average excess scattering length density of a *j*-mer of mass M(j). Equation (2) simplifies to the usual expression for a Z-average when $\overset{¯}{\rho }$ is the same for all *j*-mers (Pedersen, 1997).

### Multivalent-binding model

The multivalent-binding model of the native casein micelle has been described (Bijl et al., 2019, Holt, 2021, Holt and Carver, 2022, Lenton et al., 2020). It provides a theoretical expression for the discrete number fraction of micelles, N(j), containing *j* CaP nanocluster complexes where $j\in \{\text{1, 2, 3,}\;\text{.}\;\text{.}\;\text{. ,}\;j_{\max}\}$. The theoretical distribution is unimodal and provides a good fit to the main peak of the observed micelle size distribution, but it does not extend to fitting a tail of infrequent but large micelles (Holt et al., 1978, Saveyn et al., 2010). The distribution is specified by two parameters, $K_{\mathrm{coat}}x_{0}$ and $K_{\mathrm{core}}x_{1}$. The value of $K_{\mathrm{core}}x_{1}$ is a measure of the equilibrium between monomeric and multimeric CaP nanocluster complexes, whereas $K_{\mathrm{coat}}x_{0}$ is a measure of the equilibrium distribution of free caseins between the serum and micelle. These parameters are dimensionless but nominally can be decomposed into the product of an association constant and concentration. A third parameter $\varphi_{\mathrm{core}}$ is fixed by requiring that the micelle size distribution has the correct concentration of CaP nanoclusters to match the experimental salt partition. To model the biomimetic micelle size distribution, $K_{\mathrm{coat}}x_{0}$ was set equal to unity, its value in native cow casein micelles (Holt and Carver, 2022), and, using the experimental salt partition and the fixed parameters of the model (Holt, 2021), the value of $K_{\mathrm{core}}x_{1}$ was adjusted so that the scattering radius of gyration of the theoretical particle size distribution equalled the experimental value.

In a SANS contrast variation experiment with non-deuterated κ- and r-D-β-casein, the scattering radius of gyration varies with the protein composition of the coat and core. In r-D-B8K2 with α ∼ 0.5, the r-D-β- and κ-caseins are both in the coat and core and so the variation of the scattering radius of gyration with contrast (% D_2_O) is less than it would be in the hydrophobic colloid model where all the unlabeled protein is in the coat.

### Hydrophobic colloid model

In the hydrophobic colloid model of the casein micelle, a hydrophilic “schutzkolloid” protects the supposedly more hydrophobic core caseins from aggregating (Linderstrøm Lang, 1929). According to later developments of this idea, the surface of micelles is exclusively composed of a monolayer of κ-casein (Waugh and Talbot, 1971). This coat-core model provides a ready explanation for why renneting by the aspartate proteinase chymosin causes casein micelles to aggregate and form a gel. Chymosin cleaves off a soluble macropeptide from κ-casein exposing the supposedly more hydrophobic core. Additionally, the surface layer prevents the further growth of the more hydrophobic caseins in the interior of casein micelles and forms a steric barrier to prevent aggregation of the whole micelle (Holt and Horne, 1996, Horne, 1998, Linderstrøm Lang, 1929, Payens, 1966, Slattery and Evard, 1973, Tuinier and de Kruif, 2002, Walstra, 1990, Waugh, 1971). According to this model, casein micelle size varies inversely with the proportion of κ-casein because smaller particles have a higher surface-to-volume ratio.

Assuming all caseins occupy the same volume in the micelle, the mole fraction of κ-casein is equal to the volume fraction of coat when averaged over the size distribution.

(3) $$x_{\kappa }=\frac{4\pi t\sum_{j=1}^{j_{\max}}N\left(j\right)R_{\mathrm{core}}^{2}\left(j\right)}{4\pi t\sum_{j=1}^{j_{\max}}N\left(j\right)R_{\mathrm{core}}^{2}\left(j\right)+\left(4/3\right)\pi \sum_{j=1}^{j_{\max}}N\left(j\right)R_{\mathrm{core}}^{3}\left(j\right)}$$

Rearrangement and substitution of equation (3) gives

(4) $${\overset{¯}{R}}_{\mathrm{core},3/2}=3t\left(x_{\kappa }^{-1}-1\right)$$

In other words, the average size of the core is inversely proportional to the mole fraction of κ-casein. At $x_{\kappa }=0$ the micelle size is infinite and at $x_{\kappa }=1$ no micelles can exist. Unlike the multivalent-binding model, there is no minimum size. In some hydrophobic colloid models, there is a substructure due to protein submicelles and these effectively provide a minimum size (Slattery and Evard, 1973, Walstra, 1999) but such models are incompatible with current ideas of casein micelle substructure (de Kruif, 2014, Ingham et al., 2015, Ingham et al., 2016, Takagi et al., 2023).

In the hydrophobic colloid models, the scattering radius of gyration at the match point of the coat is that of the core: ${\left(3/5\right)}^{1/2}R_{\mathrm{core}}$ but at the match point of the core, the scattering radius of gyration has increased to a value between the core and coat radii: ${\left[3\left(R_{\mathrm{coat}}^{5}-R_{\mathrm{core}}^{5}\right)/5\left(R_{\mathrm{coat}}^{3}-R_{\mathrm{core}}^{3}\right)\right]}^{1/2}$. Thus, for the r-D-B8K2 sample with *t* = 6.7 nm and $x_{\kappa }=0.24$, equation (4) gives ${\overset{¯}{R}}_{\mathrm{core},3/2}=63.7$ nm. At the match point of the κ-casein coat (41.6 % D_2_O), the radius of gyration,${\overset{¯}{R}}_{g,3/2}=49.4$ nm and at the core match point, ${\overset{¯}{R}}_{g,3/2}=67.2$. As expected for this hydrophobic colloid model, there is a substantial dependence of the radius of gyration (in units of nm) on the scattering contrast, given by:

(5) $${\overset{¯}{R}}_{g,3/2}=37.7+28.1x_{D_{2}O}$$

## Results and discussion

### Biomimetic casein micelles, B8Kx

The hydrophobic colloid and multivalent-binding models can be distinguished by measuring the dependence of micelle size on the κ-casein concentration. We describe the preparation and properties of a range of biomimetic casein micelles of different sizes with minimal compositional complexity.

### Use of urea/urease to form B8Kx biomimetic casein micelles

**Fig. 1:**
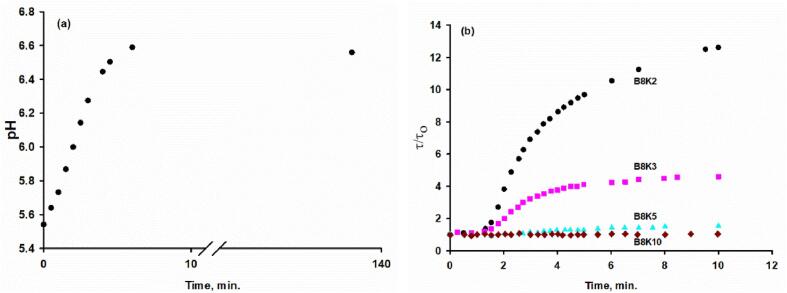
Preparation of the B8Kx biomimetic micelles using the urea/urease method to raise the pH to 6.7. (a) 5 mL of B8K3 solution at a pH of approximately 5.5 was brought up to pH 6.7 by adding 3.0 mg of urea and 195 μg of jack bean urease. The pH rose to 6.6 within 10 min. and then passed through a shallow maximum caused by equilibration of liberated CO_2_ with the atmosphere before continuing to rise slowly to the target pH over the following 24 h. (b) The change of turbidity with time of B8Kx during urea hydrolysis. The turbidity of each B8Kx solution was normalised by dividing its value (τ) by the turbidity before the addition of the urease (τ0). The biomimetic micelles assemble above pH 6.0, where the CaP nanoclusters are formed. The equilibrium size increased as the value of x in B8Kx decreased.

Examples are provided in Fig. 1 of the use of the urea/urease method to prepare biomimetic casein micelles. At the starting pH of 5.5, the caseins are soluble but no CaP can precipitate from solution because the solutions are undersaturated. The pH can be raised homogeneously, at a controlled rate and to a defined extent, by adding urea and urease to form the strong base ammonium hydroxide and the weak carbonic acid. The increase of pH initiates what would be the phase separation of CaP above about pH 6.0, but in the presence of the caseins an equilibrium complex is formed in which the CaP is in the form of nanoclusters bound to a shell of β-casein. The complexes further associate to form biomimetic casein micelles, and this produces a variable increase in the turbidity of the solution, depending on the concentration of κ-casein, as shown in Fig. 1a and 1b. In the preparation of the casein micelles, β-casein was maintained constant at 8 gL^-1^ with κ-casein being varied from 2 to 20 gL^-1^ with the concentration of CaP nanoclusters being kept constant. Hence, the nomenclature used was B8Kx, referring to β- and κ-casein respectively.

### Effect of varying x on the average size of B8Kx micelles

**Fig. 2:**
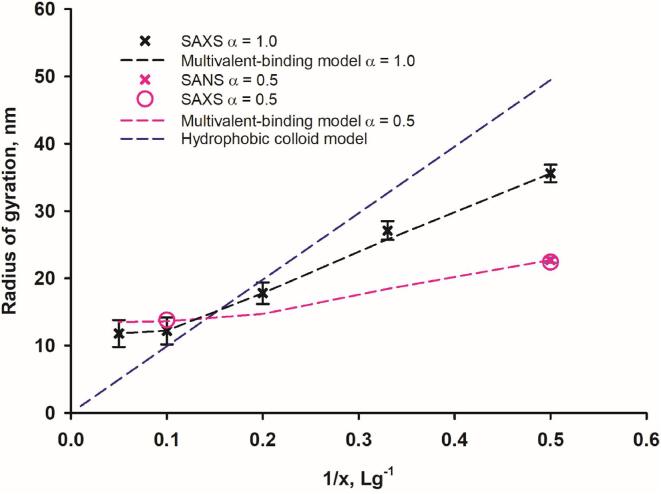
The average radius of gyration of biomimetic micelles as determined by SAXS or SANS depends on the value of x in B8Kx. Data points are radii of gyration for B8Kx from SAXS (black crosses) and SANS measurements (red crosses and red circles). The dashed lines are theoretical plots of simulations for the variation in the radius of gyration for different B8Kx mixtures, as derived from the multivalent-binding and hydrophobic colloid models of casein micelle structure. α represents the fraction of phosphoserines bound to CaP nanoclusters. (For interpretation of the references to colour in this figure legend, the reader is referred to the web version of this article.)

As the concentration of κ-casein increased, the SAXS intensity of scatter per unit of protein concentration decreased and the radius of gyration of the biomimetric casein micelle decreased (Fig. 2). Above x = 10, there was little to no further decrease in micelle size. The Guinier region was accessible for samples with x ≥ 2 (as indicated in Fig. 2).

#### Multivalent-binding model as representative of the scattering data

**Fig. 3:**
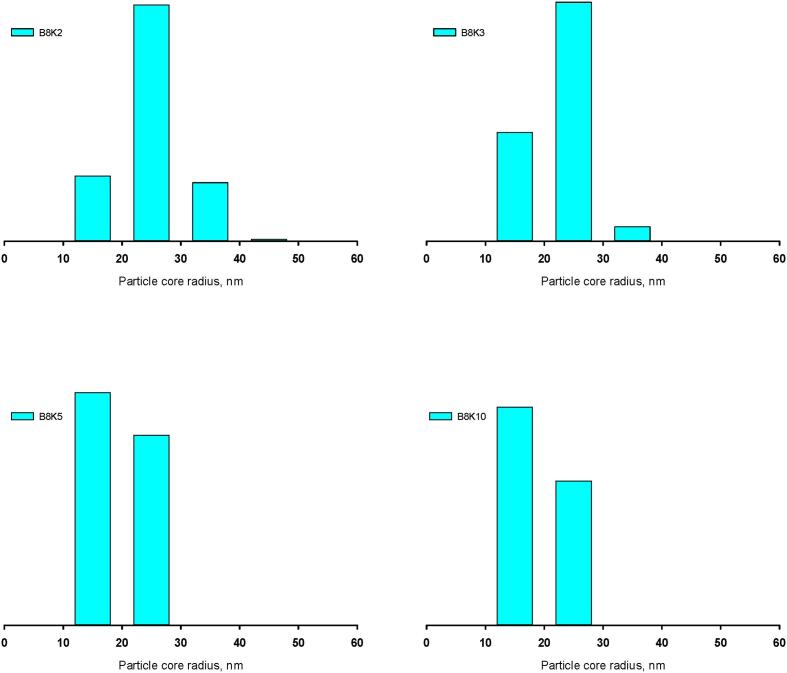
Histograms of B8Kx particle frequency versus core radii consistent with the experimental average radii of gyration. Histogram height is the total particle frequency within bins of width 10 nm. Within each histogram the total frequency was normalised to 1. The breadth of the distributions decreased as x in B8Kx increased from 2 to 10.

The dashed black and pink lines in Fig. 2 were calculated from the multivalent binding model for the two sets of samples in which the fraction of bound β-casein, α, was 1.0 or 0.5 (Holt, 2021). These calculated sizes closely matched the experimental findings and demonstrated that there was a limiting minimum micelle size at high κ-casein concentration corresponding to the radius of gyration of a single CaP-nanocluster-casein complex. Only one parameter was varied in fitting all the samples with a given value for α. Histograms of the micelle size distributions are shown in Fig. 3 for x = 2, 3, 5 and 10 in the B8Kx biomimetic micelles with α = 0.5.

As the value of x increased, i.e. at higher concentrations of κ-casein, the maxima in the calculated histograms moved to smaller core radii and the histogram breadth decreased towards the minimum size to reach nearly monodisperse distributions when x = 10.

Table 1 provides a comparison of the multivalent-binding model with experimental results for the salt partition and particle size. The value of the salt partition parameter $\varphi_{\mathrm{core}}$, was adjusted until the micelle size distribution contained the correct number of CaP nanoclusters to match the experimental concentration of non-ultrafiltered (micellar) orthophosphate. The fitted values of the self-association affinity, $K_{\mathrm{core}}x_{1}$, decreased as x in B8Kx increased. Table 1 also lists values for other calculated properties of the biomimetic micelles such as the average net charge on the free and bound caseins, the average molecular mass of the micelles and the casein composition of the coat. The molar proportion of κ-casein in the coat also varied with x from 43.3 % in B8K2 to 75.2 % in B8K10. The implication is that the incorporation of κ-casein in a protein layer around the CaP nanocluster complexes reduced the affinity of the complexes for each other and made smaller micelles, since other factors affecting casein micelle size were kept constantHydrophobic colloid model as representative of the scattering data.

According to equation (4) derived from the hydrophobic colloid model (see Materials and Methods section), the core radius of gyration of B8Kx varies inversely with the mole fraction of κ-casein. As shown by the dashed blue line in Fig. 2, the model differs from the experimental findings in two respects. Firstly, there is no minimum size of the micelle and secondly, the calculated slope is considerably steeper than that observed experimentally. The steeper slope in the hydrophobic colloid model arises because κ-casein is postulated to be located only in the coat and the coat is exclusively formed from κ-casein.

Evidence that the coat contains not just κ-casein, but also a substantial proportion of Ca-sensitive caseins has come from an analysis of the casein composition of micelles fractionated by size using either chromatography (Donnelly et al., 1984) or differential centrifugation (Dalgleish et al., 1989). This finding is qualitatively consistent with the multivalent-binding model in which the coat is formed from all free caseins (including Ca-sensitive ones) rather than just κ-casein. However, during the fractionation experiments, some re-equilibration of exchangeable caseins may have occurred.

Is κ-casein a mineral chaperone?

If the core of casein micelles contains κ-casein, as predicted by the multivalent-binding model, then it is possible that this protein can also act as a mineral chaperone. Indeed, other phosphoproteins with just one or two phosphorylated residues can bind to CaP and have significant physiological effects (George and Veis, 2008, Shaw et al., 2020, Shinomura et al., 2008, Zhang et al., 2020). However, the affinity of binding increases with degree of phosphorylation (Clegg and Holt, 2009, Pampena et al., 2004, van Kemenade and de Bruyn, 1989) so the κ-casein in the micelle may be displaced by the more highly phosphorylated, and hence stronger-binding, Ca-sensitive caseins. In experiments where cow micellar CaP was isolated after proteolytic digestion and the associated peptides analysed, CaP-SLiM-containing peptides from all the Ca-sensitive cow caseins (α_S1_, α_S2_ and β) were identified but no singly-phosphorylated phosphopeptides from any of the caseins were found (Ono et al., 1998). Thus, the recognised biological functions of κ-casein are those of steric stabilization and size regulation of the casein micelle, roles shared with the other free caseins in the micelle, and to function as a molecular chaperone to suppress inappropriate aggregation, including the formation of amyloid fibrils by other caseins.

### Effect of D_2_O on cow B8Kx biomimetic casein micelles

In preparation for a SANS contrast variation experiment with deuterated casein, the effect was measured of varying the D_2_O volume fraction on the scattering radius of gyration of two of the un-deuterated biomimetic casein micelles, namely B8K2 and the nearly monodisperse, single CaP nanocluster complexes, in B8K10. Previous studies on a CaP nanocluster complex made with the N-terminal β-casein 1–25 tryptic phosphopeptide showed little effect of replacing H_2_O with D_2_O on the partition of salts, provided the comparison was made at the same pH meter reading (Holt et al., 1998). Calculations of the effect of the solvent substitution on the scattering length densities showed there should be no significant effect of the change of solvent on the calculated radii of gyration by either SAXS or SANS unless the particles changed size or substructure.

**Fig. 4:**
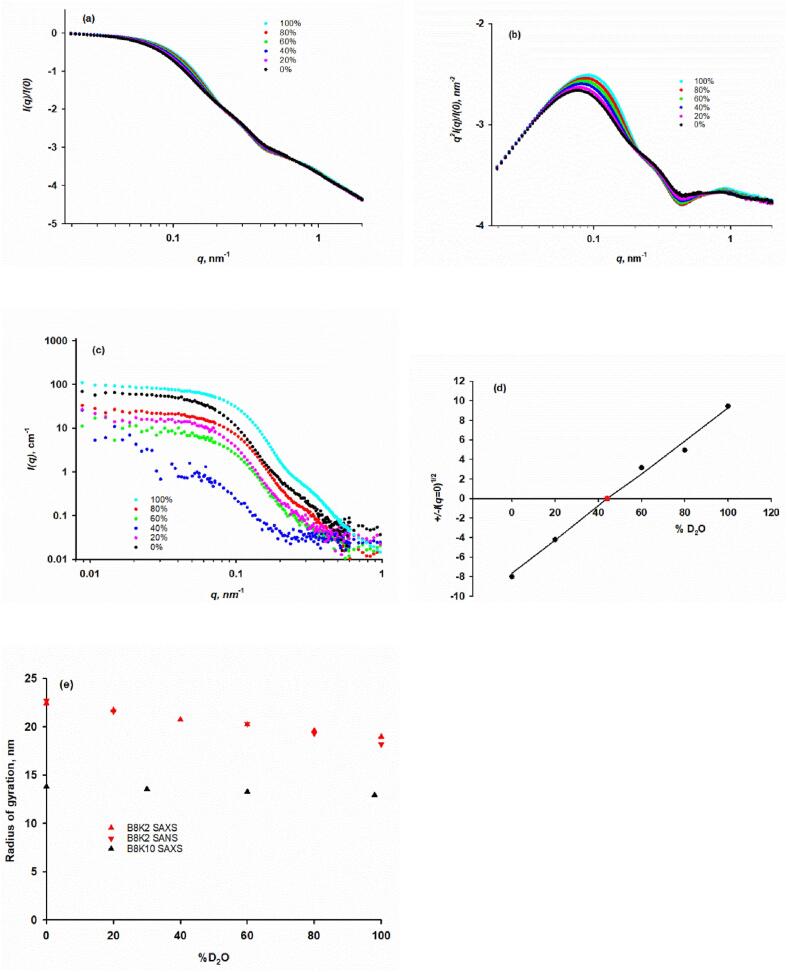
Effect of D_2_O volume fraction (v:v) on the SAXS and SANS profiles of native cow B8K2 and B8K10 biomimetic casein micelles. (a) The SAXS profiles of B8K2 in H_2_O/D_2_O mixtures exhibit two inflexion points at ∼ 0.2 and 0.5 nm^−1^ (b) SAXS q^2^-weighted Kratky plot of the B8K2 samples, confirming the shrinkage of spherical micelles with substructure produced by increasing the volume fraction of D_2_O. (c) The SANS contrast variation of B8K2 showing the reduction of scattering cross-section near the particle match point at 44 % v:v D_2_O. (d) Stuhrmann plot to determine the contrast match point by SANS of B8K2. (e) Effect of D_2_O on the SAXS and SANS scattering radii of gyration of B8K2 samples and the similar effect of D_2_O on the SAXS radius of gyration of B8K10 samples.

The SAXS profiles of B8K2 exhibit two inflexion points (Fig. 4a and the *q^2^*-weighted Kratky plot of Fig. 4b) confirming that the biomimetic casein micelles are similar to native casein micelles in being more-or-less spherical particles with substructure. They also show that micelle size and substructure are sensitive to the solvent composition. More detailed modeling studies are beyond the scope of this paper. The effect of contrast variation on the SANS profile of B8K2 is shown in Fig. 4c. The Guinier region away from the protein match point allowed the scattering radii of gyration and intercepts at *q* = 0 to be calculated. The SANS match point from a Stuhrmann plot of ± $I^{1/2}\left(q=0\right)$versus the volume fraction of D_2_O (Stuhrmann, 1974) was at 43.9 % D_2_O (Fig. 4d), in good agreement with the match point calculated from the composition of B8K2. The scattering measurements in 40 % D_2_O, shown in Fig. 4c, are therefore close to the match point and more affected by measurement noise and the effect of fluctuations from the mean scattering length density within the micelles than at other contrasts. For a chemically heterogeneous particle like the casein micelle, the fluctuations in scattering length density are particularly associated with the particle substructure of CaP nanocluster complexes, their scattering properties and their arrangement within the spherical micelle.

In changing from 100 % H_2_O to 100 % D_2_O, the scattering radius of gyration calculated from the Guinier region of both the SAXS and SANS scattering profiles of B8K2 decreased by about 10 % (Fig. 4e) along with an increase in the intercept at *q* = 0 of the SANS profile, also by about 10 %. Likewise, B8K10 had the same qualitative and quantitative changes of about 10 % in scattering radius of gyration and intercept by SAXS in comparing the profiles for H_2_O with D_2_O (Fig. 4e).

A provisional interpretation of these data is that there is a change in the solvent quality; D_2_O is not quite as good a solvent as H_2_O for the two caseins in the biomimetic micelles, and it causes a small contraction of the particles and an adjustment of the size distribution to give a modest increase in average mass. The change of molecular mass is not large enough to affect the determination of the contrast match point by means of the Stuhrmann plot shown in Fig. 3d. In a contrast variation SANS investigation of casein micelles from cow’s milk with residual fat particles (Bouchoux et al., 2015), increasing the volume fraction of D_2_O produced a small increase in the average scattering radius of gyration which is the opposite of that observed here with the simplified model system.

### Effect of deuteration of recombinant β-casein on the average size of r-D-B8K2 biomimetic casein micelles

**Fig. 5:**
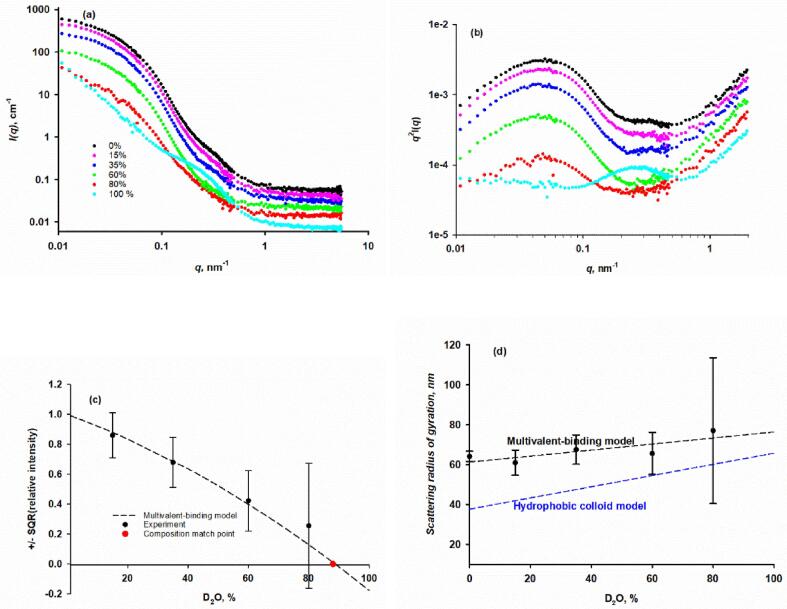
Effect of D_2_O on the SANS profile of recombinant deuterated and phosphorylated β-casein in the biomimetic casein micelle r-D-B8K2. (a) SANS profiles of r-D-B8K2 in 0 to 80 % D_2_O exhibit a short Guinier region and substructure at higher q. The radius of gyration increased slightly with increasing % D_2_O but was about three times larger than protonated B8K2 micelles. In 100 % D_2_O, r-D-B8K2 is close to its match point and was too large to calculate the radius of gyration from the restricted Guinier region, but a prominent substructural feature is present at q = 0.27 nm^−1^. (b) The q^2^-weighted Kratky plot has the shape expected of a spherical particle with substructure. The main peak decreased in amplitude and moved to lower q as the % D_2_O increased. (c) The data at the lowest q values satisfying the criterion qR_g_ < 1 were used to calculate the radius of gyration and scattering cross-section by extrapolation to q = 0. The plot of square root of relative intensity, $I^{1/2}\left(q=0\right)$, versus % D_2_O extrapolated to a match point of 105 ± 6.5 % D_2_O, a value larger than the match point of the particles calculated from their composition of 88.7 %. (d) The experimental radii of gyration as determined from SANS measurements. The data do not fit the hydrophobic colloid model but are fitted by the multivalent-binding model after allowing the micelles to become slightly larger with increasing % D_2_O.

The results of a SANS contrast variation experiment on biomimetic casein micelles with x = 2, formed with unlabelled cow κ-casein, CaP nanoclusters and recombinant, highly deuterated and phosphorylated β-casein to give the sample r-D-B8K2 are presented in Fig. 5. The SANS intensity varied with % D_2_O, reflecting the match point calculated from the composition of 88 % for the deuterated micelles (Fig. 5a). In H_2_O, the substructure inflexion is at *q =* 0.27 nm^−1^. In 80 % D_2_O, close to the match point for the CaP, the substructure is hardly seen but at 100 % D_2_O, close to the match point of the deuterated β-casein, the scattering is strongly influenced by the CaP substructure and the κ-casein. The scattering at low *q* in H_2_O and D_2_O indicates that the deuterated micelle is much larger than the corresponding protonated B8K2 (Fig. 5a) as judged by the small number of data points falling in the Guinier region in H_2_O. These become fewer as the % D_2_O increases until there is effectively no Guinier region in 100 % D_2_O. Thus, the micelles show an increase in size with an increase in % D_2_O. The Kratky plot in Fig. 5b confirms that the deuterated micelles are spherical particles with substructure and the movement of the main peak to lower *q* as the % D_2_O increases confirms that the particle size increased with the change of solvent from H_2_O to D_2_O. The intercepts were normalised by dividing by the intercept in H_2_O and are shown as a type of Stuhrmann plot in Fig. 5c. If the particles did not change their size with the solvent, then the Stuhrmann plot in Fig. 5c would be linear and extrapolation of the experimental values to zero would give a calculated match point near 105 % D_2_O, much larger than the match point calculated from the composition. The intercepts at *q* = 0 and scattering radii of gyration (Fig. 5d) were calculated from the experimentally accessible Guinier region. The radius of gyration of r-D-B8K2 in H_2_O is about three times larger than the B8K2 micelles in H_2_O and increased by a small amount from 64.1 ± 2.6 nm in H_2_O to 65.6 ± 10.5 nm in 80 % D_2_O. The relatively large measurement error in 80 % D_2_O is mainly due to the small size of the Guinier region.

The data in Fig. 5 were fitted to the multivalent-binding model and the results are shown in Table 2. The larger size of the r-D-B8K2 micelles compared to the B8K2 micelles is easily accounted for by this model. The principal factors are a higher proportion of bound r-D-β-casein, which causes the particle size to increase. In B8K2, α = 0.57 which increased to 0.62 in r-D-B8K2. A second important effect is the larger self-association affinity, *K_core_x*_1_, indicating a greater tendency of the CaP nanocluster complexes made from the deuterated β-casein to associate and form larger micelles. For the B8K2 micelles in H_2_O, $K_{\mathrm{core}}x_{1}=0.84$, which was increased to 0.992 to fit the size of r-D-B8K2 micelles in H_2_O and 0.997 in 80 % D_2_O. This adjustment produced an excellent agreement between the model and experiment. Thus, there was a linear increase of radius of gyration with increasing % D_2_O (black dashed line in Fig. 5d) and the relative intensity decreased with % D_2_O (curved black dashed line in Fig. 5c). The curved line intercepted the axis at zero relative intensity, to give a calculated match point of 87 % D_2_O, very close to the match point of 88.7 % D_2_O calculated from the composition (See supplementary Table 1).

**Table 2:** Calculated effect of D_2_O on the average size and intensity at q = 0 of the biomimetic r-D-B8K2 casein micelles in H_2_O-D_2_O mixtures using either the multivalent-binding or hydrophobic colloid models.

	**% D_2_O**					
	0	20	40	60	80	100
**Multivalent-binding model**						
Radius of gyration, nm	61.3	64.3	67.3	70.3	73.4	76.4
$I_{q=0}\left({\text{H}}_{\text{2}}\text{O}\right)/I_{q=0}\left(\%D_{\text{2}}\text{O}\right)$	1.00	0.697	0.405	0.159	0.017	−0.031
$K_{\mathrm{core}}x_{1}$	0.9918	0.9926	0.9939	0.9946	0.9954	0.9959
**Hydrophobic colloid model**						
Radius of gyration, nm	37.7	43.3	48.9	54.5	60.1	65.8

The hydrophobic colloid model does not provide an explanation for why r-D-B8K2 micelles are three times bigger than B8K2 micelles since, according to this model, the size is solely determined by the mole fraction of κ-casein. Indeed, because of the higher molecular mass of the deuterated β-casein, the mole fraction of κ-casein is higher in r-D-B8K2 at 0.315 compared to 0.300 in B8K2. Thus, micelles made from the deuterated β-casein should be slightly smaller, not three times larger, if the hydrophobic colloid model were operational. The hydrophobic colloid model also predicts a steeper dependence of the SANS radius of gyration on % D_2_O than is observed experimentally (Fig. 5d) due to the hydrophobic colloid model, unlike the multivalent-binding model, having no deuterated β-casein molecules in the coat and no protonated κ-casein in the core.

The intrinsically disordered nature of casein proteins is much more compatible with the multivalent-binding model of the casein micelle compared to the hydrophobic colloid model (Holt, 2021, Holt and Carver, 2022). An IDP-containing model readily explains why caseins are amyloid fibril-forming proteins and have molecular chaperone properties comparable to the small heat-shock proteins in preventing the aggregation of unfolding proteins (Bhattacharyya and Das, 1999, Morgan et al., 2005). The hydrophobic effect is too small in the intramolecular interactions of IDPs to cause conformational collapse of caseins, so how can it be the dominant force in their intermolecular interactions? A statistical mechanical analysis of several types of casein association reactions (Harton and Shimizu, 2019) showed the minor importance of the hydrophobic effect compared to electrostatic interactions. Sequence analysis using reliable scales of amino acid residue hydrophobicities (Holt et al., 2019) and the lack of conservation of hydrophobic residues in aligned sequences (Manguy and Shields, 2019) also indicate that caseins are not hydrophobic proteins. Furthermore, caseins have the characteristic amino acid composition of IDPs [91]. In agreement with these arguments, the experimental evidence presented here clearly favours the multivalent-binding model over the hydrophobic colloid model as representative of the arrangement of caseins within the micelle.

### Structure and functions of casein micelles compared to the calciprotein particles of blood

We have shown here that the multivalent-binding model of the casein micelle provides a much better fit to the properties of biomimetic casein micelles than hydrophobic colloid models in which the κ-casein is exclusively found in a surface coat. Indeed, we suggest that the proposed concentration of the most amyloidogenic casein in the surface of the hydrophobic colloid models is likely to favour the growth of amyloid fibrils and may prevent the other caseins, acting as molecular chaperones, from binding to the κ-caseins to prevent amyloid fibril formation. In the multivalent-binding model, the κ-casein is in intimate contact with the other caseins which can therefore inhibit the formation of amyloid fibrils.

Casein micelles contain about 1.5 x 10^19^ CaP nanocluster complexes with casein per litre of cow milk (Holt and Carver, 2022); about 10 orders of magnitude larger than is estimated for blood. As a subject for the study of the action of mineral chaperones on biofluid stability, milk has the advantages of ready availability, but with a simpler and better-defined composition than either blood or the extracellular matrix. Moreover, precise and highly detailed physico-chemical models of the salt and protein chemical equilibria in milk are available (Bijl et al., 2019, Holt, 2021, Lenton et al., 2020).

The most significant difference between the milk CaP nanocluster complexes with the Ca-sensitive caseins and the blood calciprotein particles is their stability. The blood calciprotein particles mature from type I to type II within hours or days (Jahnen-Dechent and Smith, 2020, Jahnen-Dechent et al., 2020) whereas the CaP nanocluster complexes are indefinitely stable, reflecting the need to store milk in the cisterns and ducts of the mammary gland for weeks or even months, depending on the reproductive strategy of the species (Power and Schulkin, 2016). In terms of reaction kinetics, the activation energy barrier opposing maturation of amorphous CaP into a more crystalline phase increases as the concentration of mineral chaperone increases (de Bruyn et al., 2013, Lenton et al., 2020). In the quasi-equilibrium initial state of a core–shell complex, some insight into the reasons for stability was gained by applying equilibrium thermodynamics to calculate the equilibrium core radius of the CaP (Holt, 2013). As a result, it was clear that the free energy of binding of a mineral chaperone to a crystalline phase would be too small to form a stable nanoparticle because of the large lattice energy of crystals would drive the formation of a macroscopic crystalline phase. However, an equilibrium or metastable core–shell nanoparticle was possible if the core was an amorphous and highly hydrated form of CaP. Likewise, stability can be improved by increasing the affinity of the sequestering phosphoprotein for the CaP and by decreasing its footprint on the core surface with tight packing. In respect of the latter factor, flexible IDPs containing types of CaP-SLiMs may be advantageous over more rigid globular proteins. These considerations may prove useful in the design of biomimetic mineral chaperones for remedial or preventative treatments of ectopic and pathological calcification.

## Funding

This work was supported by the ANSTO Australian Synchrotron proposal P9858, and ANSTO Australian Centre for Neutron Scattering proposals P4288, P6146 and ANSTO National Deuteration Facility proposals NDF2878, NDF3516, NDF3915 and NDF5725.

## CRediT authorship contribution statement

**Jared K. Raynes:** Conceptualization, Data curation, Funding acquisition, Investigation, Writing – original draft, Writing – review & editing. **Jitendra Mata:** Writing – review & editing, Resources, Methodology, Investigation. **Karyn L. Wilde:** Writing – review & editing, Resources, Methodology, Investigation. **John A. Carver:** Writing – review & editing, Funding acquisition, Conceptualization. **Sharon M. Kelly:** Resources, Methodology, Investigation. **Carl Holt:** Writing – review & editing, Writing – original draft, Methodology, Investigation, Formal analysis, Conceptualization.

## Declaration of competing interest

The authors declare the following financial interests/personal relationships which may be considered as potential competing interests: Jared K. Raynes undertook this research whilst based at the CSIRO but is now Chief Scientific Officer at All G Foods which is making biomimetic casein micelles from recombinant caseins. Carl Holt is a Scientific Advisor to All G Foods.

## Data Availability

Data will be made available on request.
