# Supplementary material for: Structure of biomimetic casein micelles: Critical tests of the hydrophobic colloid and multivalent-binding models using recombinant deuterated and phosphorylated β-casein
Source: J Struct Biol X. 2024 Jan 22;9:100096. doi: 10.1016/j.yjsbx.2024.100096 (PMC10840362; doi:10.1016/j.yjsbx.2024.100096)
Supplement: Supplementary Data 1 [file mmc1.docx]

# Supplementary Materials

# Structure of Biomimetic Casein Micelles: Critical Tests of the Hydrophobic Colloid and Multivalent-Binding Models Using Recombinant Deuterated and Phosphorylated β-Casein

## Jared K. Raynes*^1,2^, Jitendra Mata^3^, Karyn L. Wilde^4^, John A. Carver^5^, Sharon M. Kelly^6^ and Carl Holt^6^

1. ***CSIRO Agriculture & Food****, 671 Sneydes Road, Werribee, VIC 3031, Australia. (*[*https://orcid.org/0000-0002-8252-5980*](https://url.avanan.click/v2/___https://orcid.org/0000-0002-8252-5980___.YXAzOmFsbGdmb29kczphOm86MGRiYjU1OTVhMTI4OWEyNjViMjkyM2ExNjk4YmIzOWI6Njo1ZjVlOmYyZDY5NmM1MzY5NDlkYjJmNGIyNzNiMjJjODRlNTJmYTljN2IwMDQ5YTI4ZGUwZDRkMjU4NzJmZWRmMzUxOWE6cDpU)*)*
2. ***All G Foods****, Waterloo, NSW 2006, Australia.(jared@allgfoods.com,*[*https://orcid.org/0000-0002-8252-5980*](%20https://orcid.org/0000-0002-8252-5980)*)*
3. ***Australian Centre for Neutron Scattering****, Australian Nuclear Science and Technology Organisation, Lucas Heights, NSW 2234, Australia. (*[*https://orcid.org/0000-0001-9225-7900*](https://url.avanan.click/v2/___https://orcid.org/0000-0001-9225-7900___.YXAzOmFsbGdmb29kczphOm86MGRiYjU1OTVhMTI4OWEyNjViMjkyM2ExNjk4YmIzOWI6NjoxOWQ4OjYzNzQ0MDMzNzhhMzE3YzhiOGY4MGM2NzU2ZDFjNmE1ZWRiZWM5MmEzYjExMDA1OTczZDFhNDI4MzhhN2MyMmY6cDpU)*)*
4. ***National Deuteration Facility****, Australian Nuclear Science and Technology Organisation, Lucas Heights, NSW 2234, Australia. (*[*https://orcid.org/ 0000-0002-0538-6796*](https://url.avanan.click/v2/___https://orcid.org/%200000-0002-0538-6796___.YXAzOmFsbGdmb29kczphOm86MGRiYjU1OTVhMTI4OWEyNjViMjkyM2ExNjk4YmIzOWI6NjplMzAzOmM2YmVhOWFmYjg2NDUxN2IwMzdmMzY3NjViZjc3YjUxZmRhNDZjMWMyOTIyNzljNGRkOGM0MDdkN2QyYzI3YWQ6cDpU)*)*
5. ***Research School of Chemistry****, The Australian National University, Acton, ACT 2601, Australia. (*[*https://orcid.org/0000-0002-2441-8108*](https://url.avanan.click/v2/___https://orcid.org/0000-0002-2441-8108___.YXAzOmFsbGdmb29kczphOm86MGRiYjU1OTVhMTI4OWEyNjViMjkyM2ExNjk4YmIzOWI6NjoxNzFmOjI0OTlhZmQ3NzIzOTBkNTgwNmYxNTVhMTk4YWRjNmYzMjkzNzFkN2ExYjBiY2U5YzFhNjk2YmRkYjg0M2I0Mzk6cDpU)*)*
6. ***School of Molecular Biosciences****, University of Glasgow, Glasgow G12 8QQ, United Kingdom. (*[*https://orcid.org/0000-0002-3516-1387*](https://url.avanan.click/v2/___https://orcid.org/0000-0002-3516-1387___.YXAzOmFsbGdmb29kczphOm86MGRiYjU1OTVhMTI4OWEyNjViMjkyM2ExNjk4YmIzOWI6NjphYTVhOjQ0ZDNhZGQ4ZDM3MWE2ZmM4OGE0ZDQzY2UyMDNkYmJmMzg2YzI1N2ZmZDdhNDJkZGE5N2YyYjY3Zjk0ODUxNjQ6cDpU)*) and (*[*https://orcid.org/0000-0002-2087-1546*](https://url.avanan.click/v2/___https://orcid.org/0000-0002-2087-1546___.YXAzOmFsbGdmb29kczphOm86MGRiYjU1OTVhMTI4OWEyNjViMjkyM2ExNjk4YmIzOWI6NjpkMTI5OmEzNjJmYWFlMzI5ZjAzZWE0OGI0ZjFiZGUxZmRjOGJmMzYxYmU4MWIxODA3YjMwNTMwMjc4MzgyOTMzOWMzZmM6cDpU)*)*

****Correspondence to Jared K. Raynes. All G Foods Limited.***

## Section 1. Scattering length density calculations

Scattering length densities (SLDs) were calculated for all the components of the biomimetic B8Kx casein micelles and are summarised in Table 1.

### Table 1. Scattering length densities (SLD) (10^-4^nm^-2^) of components of the biomimetic B8Kx micelles

| Sample | Empirical Chemical Formula | Mass | Volume | Neutron SLD | | Match Point | X-ray SLD | |
| --- | --- | --- | --- | --- | --- | --- | --- | --- |
|  |  | Da | nm^3^ | H_2_O | D_2_O | % D_2_O | H_2_O | D_2_O |
| β-CN 5-P | C1080H1370N268O325S6P5 | 23,927.5 | 30.80 | 1.695 | 2.738 | 38.2 | 11.56 | 11.42 |
| κ-CN 1-P | C849H1324N223O265S4P1 | 19,011.1 | 24.17 | 1.792 | 3.094 | 41.6 | 11.65 | 11.46 |
| r-D-β-CN 4-P | C1074H485D1188N269O331S7P4 | 25,185.8 | 30.59 | 5.795 | 6.763 | > 100 | 12.14 | 11.99 |
| CaP | Ca(HPO_4_)_0.4_(PO_4_)_0.4_ | 116.45 | 0.06382 | 4.057 | 4.694 | 86.5 | 25.97 | 25.89 |
| H2O | H2O |  |  | -0.55 |  |  | 9.4 |  |
| D2O | D2O |  |  |  | 6.35 |  |  | 9.4 |

Amino acid sequences of the mature proteins (i.e., without the signal sequence) were taken from the UniprotKB website ([https://www.expasy.org/resources/uniprotkb-swiss-prot](https://url.avanan.click/v2/___https://www.expasy.org/resources/uniprotkb-swiss-prot___.YXAzOmFsbGdmb29kczphOm86MGRiYjU1OTVhMTI4OWEyNjViMjkyM2ExNjk4YmIzOWI6NjpiYTQ2OmM1MTg0YTZlMTAzNzcyOGYwYmMxM2U3M2I4MzEzYTliZWRlYjYzZDVlNTdiYWExNDUxYzU2MGMyZGI5OWY5MGQ6cDpU)) to give the elemental composition after allowing for average levels of phosphorylation of 1, 5, 8 and 12 for κ-, β-, α_S1_- and α_S2_-caseins, respectively . The average scattering length density of particles, $\bar{\rho}_{x_{D_{2}O}}$, in a solvent with a D_2_O volume fraction, $x_{D_{2}O}$, was calculated from the average scattering length densities of the two caseins and the CaP in the nanoclusters, weighted by their mole fractions in the micelle, using

$$\bar{\rho}_{x_{D_{2}O}}=\bar{\rho}_{H_{2}O}+(\bar{\rho}_{D_{2}O}-\bar{\rho}_{H_{2}O})x_{D_{2}O}$$

Protein molecular mass and volume and the number of exchangeable protons were calculated from their amino acid composition using the tabulated values of (Jacrot and Zaccai, 1981). Each phosphorylation of an amino acid residue added 80 Da to the molecular mass and 0.0588 nm^3^ to the molecular volume. The chemical composition of the CaP in complexes with casein phosphopeptides was obtained from the experimental invariant solubility product in citrate-free media in the pH range 6.1 – 6.8 (Little and Holt, 2004). All the protons in the empirical chemical formula, $\text{Ca(HPO}_{4}\text{)}_{0.4}\text{(PO}_{4}\text{)}_{0.4}$, were assumed to be fully exchangeable.

## Section 2. SANS data acquisition

### Table 2. SANS experimental parameters

| Volume fraction D_2_O | Sample to Detector Length (m) | Neutron Wavelength (nm) | Sample exposure Time (sec) | Optical path length (mm) |
| --- | --- | --- | --- | --- |
| 0 | 1.3 | 5 | 900 | 1 |
| 0 | 12 | 5 | 1800 | 1 |
| 0 | 20 | 8.1 | 3600 | 1 |
| 0.15 | 1.3 | 5 | 900 | 1 |
| 0.15 | 12 | 5 | 1800 | 1 |
| 0.15 | 20 | 8.1 | 3600 | 1 |
| 0.35 | 1.3 | 5 | 900 | 1 |
| 0.35 | 12 | 5 | 1800 | 1 |
| 0.35 | 20 | 8.1 | 3600 | 1 |
| 0.6 | 1.3 | 5 | 900 | 2 |
| 0.6 | 12 | 5 | 1800 | 2 |
| 0.6 | 20 | 8.1 | 3600 | 2 |
| 0.8 | 1.3 | 5 | 900 | 2 |
| 0.8 | 12 | 5 | 3600 | 2 |
| 0.8 | 20 | 8.1 | 3600 | 2 |
| 1 | 1.3 | 5 | 1800 | 2 |
| 1 | 12 | 5 | 3600 | 2 |
| 1 | 20 | 8.1 | 7200 | 2 |

## Section 3. Characterisation of recombinant β-casein

### Temperature-dependent self-association of cow native and recombinant β-caseins

Cow β-casein associates reversibly and endothermically as the temperature is increased from 4-40 °C to form oligomers of increasing size (Andrews et al., 1979). The endothermic process may be the result of a conformational change, as detected by circular dichroism (CD) spectroscopy (Qi et al., 2005). This interpretation is supported by endothermic association of β-casein with other caseins, including the casein micelle (Portnaya et al., 2016). Differential scanning calorimetry (DSC)Thermograms were collected in the temperature range 5 - 120°C at a protein concentration of 5 gL^-1^ as described previously (Syme et al., 2002). It was confirmed that the native cow β-casein showed an endothermic peak with a maximum at 16.2 °C (Qi et al., 2004) (Figure S1). The recombinant protein also showed an endothermic peak but at the lower temperature of 8.2 °C and with an estimated amplitude more than twice as large.


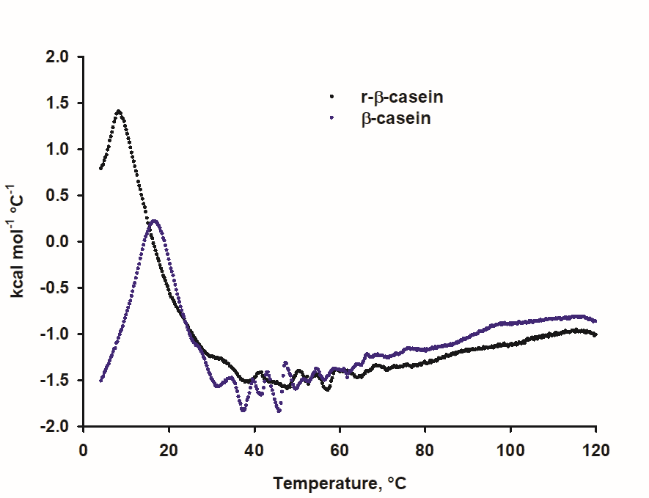


### Figure S1 Solvent subtracted thermograms of cow and recombinant β-caseins, recorded on a Microcal MCS differential scanning calorimeter, as described previously (Holt et al., 2009).

The self-association of cow or human β-casein near neutral pH is much reduced by ablation of residues at the C-terminus (Berry and Creamer, 1975; Bu et al., 2004; Farrell et al., 2001; Qi et al., 2004) and the endothermic process ascribed to the hydrophobic effect (Andrews et al., 1979; O'Connell et al., 2003). Notwithstanding this, the stronger and more endothermic self-association of the recombinant protein, which has been modified only near the N-terminus, strongly suggests that the interactions leading to association are multivalent in character and also involve the polar residues near the N-terminus.

### Circular Dichroism spectroscopy

Far-UV circular dichroism (CD) spectra were recorded with a JASCO J-810 spectropolarimeter at 1 mg mL^-1^ with a 0.2 mm path length quartz cuvette at 25 °C. Three scans, recorded at a scan rate of 20 nm per minute, were averaged for each sample.

Representative far-UV CD spectra of the starting protein mixture, B8K10 at pH 6.7, and the protein mixture adjusted to the same pH after adding calcium chloride to form CaP nanoclusters and the biomimetic micelles are shown in Figure S2.


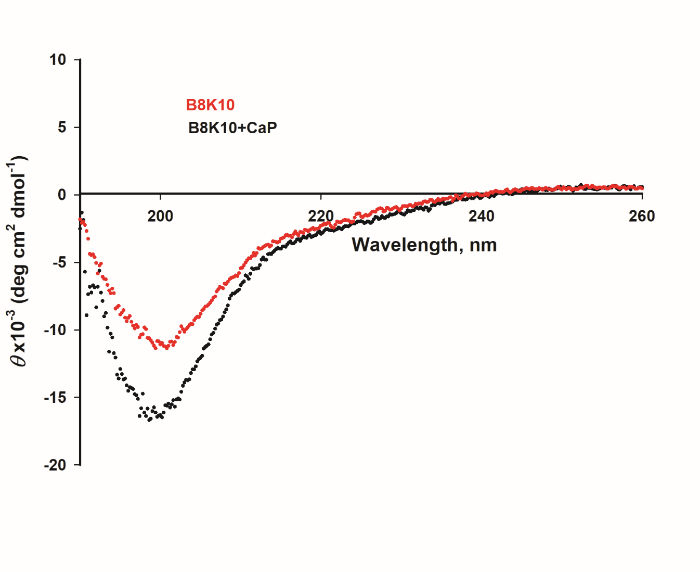


### Figure S2 Far-UV circular dichroism spectra of the cow B8K2 casein mixture in 6.5 mM phosphate buffer, pH 6.7, before and after the addition of 7.1 mM calcium chloride

The far-UV CD spectra of caseins and the biomimetic casein micelle in Figure S2 are consistent with the unfolded conformation of caseins, including the formation of poly-L-proline-II (PP-II) helical secondary structure (Syme et al., 2002), under both conditions. The amplitude of the minimum in ellipticity near 200 nm is greater in the micellar form. To explain this, we follow the interpretation of the CD spectra of random coil homopolypeptides proposed originally by (Drake et al., 1988) whereby their conformations are a mixture of PP-II and a more random structures with a very low-amplitude CD spectrum. The equilibrium between these conformations is highly temperature-sensitive because the PP-II conformation is not stabilised by hydrogen bonding but by weaker dipolar forces between backbone carbonyl and N-H polar bonds (Lazaridis and Karplus, 1999; Maccallum et al., 1995). Thus, the PP-II proportion may be higher in the biomimetic casein micelle because of a stabilising effect on the conformation due to the multivalent interactions of caseins with each other and the CaP nanocluster. The quantitative interpretation of CD spectra in terms of regular secondary structures and less ordered structures remains problematic and is not attempted here (Greenfield, 2006; Miles et al., 2021).

The method of choice for characterising the secondary structure in highly flexible IDPs such as the caseins is Raman optical activity (ROA) because the chiral effect is largely confined to a single peptide bond and the information content of spectra is high compared to far-UV CD spectroscopy or the nearly smooth amide I band of infrared spectra (Barron et al., 2002; Tompa, 2012). The predominant conformation in β- and κ-caseins is the PP-II helix according to ROA (Syme et al., 2002) with both proteins containing little or no α-helix near neutral pH. When far-UV CD spectra of caseins and many other IDPs are acquired, they invariably have the characteristic spectrum of the PP-II conformation with an ellipticity minimum close to 200 nm (Shi et al., 2006; Woody, 2009). However, when such spectra are analysed using a basis set of spectra from predominantly or exclusively globular proteins, a substantial fraction of residues is predicted to be in α-helices or other regular secondary structures, especially β-strands. The possible cause of the discrepancy (Greenfield, 2006) may be the low content of the recognised PP-II conformation in globular proteins and the difficulty in identifying a standard spectrum for PP-II to correct for this bias.

## Section 4. Preliminary characterisation of biomimetic casein micelles

### Small-angle scattering of native and biomimetic casein micelles

Small-angle X-ray and neutron scattering (SAXS and SANS, respectively) of the native casein micelle from raw milk (Holt et al., 2003; Marchin et al., 2007) has an inflexion near *q* = 0.35 nm^-1^ largely due to its substructure of CaP nanoclusters (Bouchoux et al., 2015; Holt et al., 2003; Ingham et al., 2015). At this point the scattering from the whole particle has declined to the point where it is comparable to that from the substructure and hence the position of the inflexion is affected by many factors including the whole particle size and the scale of the substructure.

Preliminary experiments on the B8Kx samples were carried out on the SAXS beamline 2.1 at the Daresbury laboratory, UK, using a camera length of 8 m, a quadrant detector and *q* calibration from the fibre diffraction peaks of wet rat tail collagen. These, and other operating conditions are close to those reported for the CaP nanocluster complexes formed with β-casein 1-25 (Holt et al., 1996).The in-house software was used for data reduction (Holt et al., 1996; Papiz et al., 2019).

### Effect of dilution

The dilution buffer has a composition that matches the degree of saturation with respect to the CaP nanoclusters in the biomimetic casein micelles and hence caseins that are directly bound to the CaP are not able to dissociate upon dilution with the buffer. Dissociation of the free caseins from the micelle is, nevertheless, possible. Dissociation of the free caseins can be reduced by increasing the free calcium ion concentration. In practice a free calcium ion concentration of 2 mM was found to be sufficient to suppress dissociation of the free caseins (Holt et al., 1986). The B8K10 system has the highest concentration of free caseins but, as shown by SAXS measurements, the dissociation upon dilution was very limited in the concentration range of interest (Figures S3a and S3b). Similar studies with the B8K2 sample showed no effect of dilution on the calculated radius of gyration or reduced scattering intensity at *q* =0 in the protein concentration range 2 – 10 gL^-1^ where measurements were usually made.







### Figure S3 Effect of dilution on (a) the radius of gyration and (b) reduced scattering intensity at q=0 of B8K10 biomimetic casein micelles.

### SAXS profile of the B8K10 biomimetic casein micelles

The preliminary experiments demonstrated that biomimetic B8K10 micelles with the calculated fraction of bound β-casein, α, close to unity were much smaller than native micelles with a scattering radius of gyration of 13.73±0.22 nm (Figure S3a). Increasing the κ-casein concentration to 20 gL^-1^ (B8K20) gave only slightly smaller particles indicating that there was a minimum size accessible by increasing the concentration of κ-casein. The SAXS profile of the biomimetic B8K10 micelles (Figure S4a) showed a Guinear region at low *q* and evidence of substructure at higher *q*. The CaP nanocluster complexes prepared with the highly acidic tryptic phosphopeptides, β-casein 4P 1-25 and α_S1_-casein 5P 59-79, each containing a single CaP-SLiM, showed no tendency to form oligomers under the conditions reported (Holt et al., 1996; Holt et al., 1998; Little and Holt, 2004). The sequence 26-209 in β-casein is a type of Pro- and Gln-rich polar tract of low charge density. Similar polar tract sequences promote self-association by IDPs (Mao et al., 2010). Thus, it is likely that the polar tract sequence in whole β-casein has increased the tendency to self-association of the B8Kx CaP nanocluster complexes.

After *q*^2^ weighting of the intensity a Kratky plot (Figure S4b) showed that the particles are spherical rather than coils or rods and that they have a substructure, as shown by a subsidiary maximum close to *q* ~ 0.3 nm^-1^. Studies on native casein micelles have shown that the substructures generate an interference effect which can be fitted by a liquid-like interparticle structure factor (de Kruif, 2014). For the much smaller B8K10 particles, the interference effect is likely to be a simple function of only two or three centres of substructure with the same nearest neighbour distance of closest approach of about 18 nm (Holt et al., 2003).


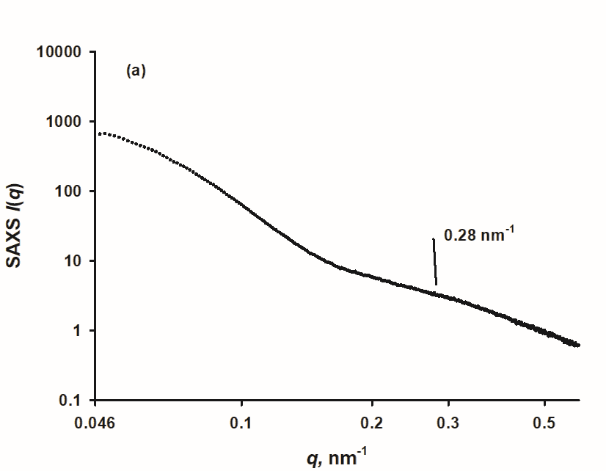

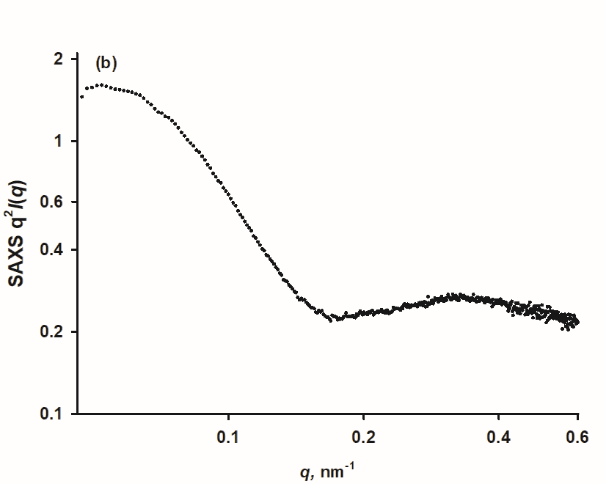


### Figure S4. Scattering profiles of B8K10 with α ~ 1.0. (a) SAXS profile of B8K10 at pH 6.7 showing the Guinier region and an inflexion at higher *q* due to substructure. (b) Kratky plot of the same data demonstrating that the particles are spherical rather than coils or rods. The primary maximum is at 0.052 nm^-1^, with a subsidiary maximum around *q* ~ 0.3 nm^-1^. For these small particles- essentially one or two CaP nanocluster complexes, the structure factor modifying the form factor of the complexes is close to unity over the measured *q* range.

### Effect of pH or 10% ethanol on the substructure of the biomimetic B8K3 micelles

Modelling of the ion equilibria in the B8Kx samples by the method of (Bijl et al., 2019) showed that as the pH was raised by the urea/urease reaction, the formation of the CaP nanoclusters began at pH 6.4 and was virtually complete by pH 7.4. The dry mass fraction of CaP in the biomimetic B8Kx micelles with α 0.5 is 0.031, which is less than half that in cow casein micelles. Using the approximate expressions for predicting the position of the inflexion of (Holt et al., 2003), the average distance between nearest neighbour CaP nanoclusters, *λ*, is 22.6 nm and the position of the major inflexion is expected around $q\approx0.28$ nm^-1^.

Figure S5a shows the changing shape and intensity of SAXS profiles as the pH was raised in a B8K3 sample. The SAXS profiles show a pH-dependent association but at pH 7 the biomimetic B8K3 nanoclusters are clearly larger than the B8K10 particles of Figures S3 and S4, demonstrating the inverse relationship of particle size on k-casein concentration. Moreover, the interference effect of their substructure is more complex in the B8K3 particles than in B8K10. Near *q* = 0.3 and 0.5 nm^-1^, the effect of micelle substructure can be observed above pH 6.0 but not below this pH where there are no CaP nanocluster complexes.


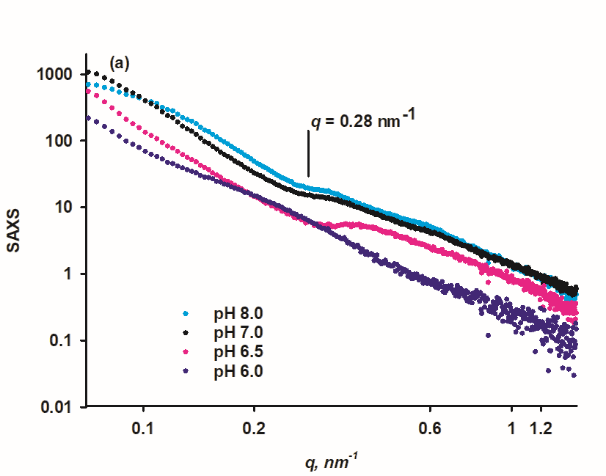

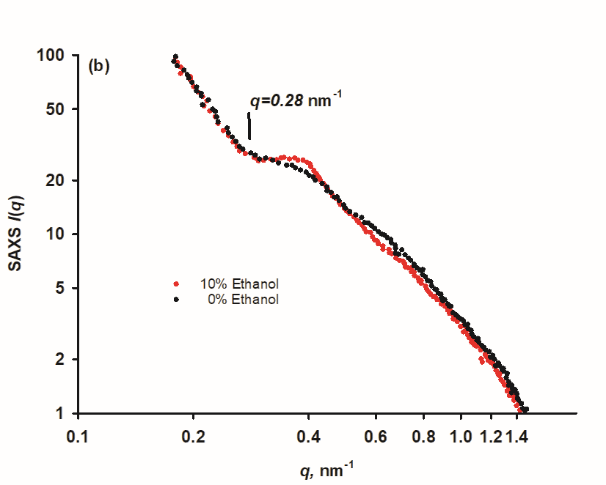


### Figure S5. SAXS profiles of B8K3 with α ~ 1.0, acquired on Daresbury beamline 2.1 with measurement parameters similar to those previously reported (Holt et al., 1996) and a camera length of 3 m. (a) The effect of pH on the SAXS profile, particularly around *q* ~ 0.28 and 0.53 nm^-1^ at pH 7 and 8 due to the substructure due to the CaP nanoclusters, but not at pH 6 where no CaP nanoclusters are present. (b) Enhanced amplitude of the substructure effect around *q*=0.3 in the SAXS profile of B8K3 micelles at pH 6.7 in the presence of 10% v:v ethanol.

Somewhat similar observations have been made of the prominent inflexion in the SAXS of native cow casein micelles, albeit at slightly higher *q* values, where lowering the pH, or adding EDTA to genipin cross-linked micelles, dissolved the CaP nanoclusters and produced an apparently more homogeneous particle (Hansen et al., 1996; Liu et al., 2017; Marchin et al., 2007; Pignon et al., 2004; Singh et al., 2020). These experiments and others (Holt et al., 2003; Ingham et al., 2015) have confirmed that the substructure effect on the SAXS spectra of native casein micelles near *q* ~ 0.3 nm^-1^ arises from interference between CaP nanocluster complexes.

Another similarity between the biomimetic and natural casein micelles is the strengthening of the substructure features by the addition of the non-solvent ethanol to the point where a subsidiary maximum may be observed at *q*~ 0.3 nm^-1^ (Figure S5b). Similar observations of the effect of ethanol on the structure of native micelles have been made by SAXS on cow and some non-bovine micelles (Day et al., 2017; Smyth et al., 2004) and in cow casein micelles by contrast variation SANS (Holt et al., 2003). What may be concluded from these comparisons is that the biomimetic and the larger native casein micelles have a similar substructural feature in the range *q* = 0.25 - 0.35 nm^-1^ and that this arises primarily from the interference effects of a substructure of CaP nanocluster complexes.

### SAXS and SANS on B8K5 biomimetic micelles and their stability to D_2_O

Triplicate SAXS measurements on B8K5 biomimetic micelles in the aqueous Mg-containing buffer with α = 1 confirmed that the particles were of the expected size and substructure (Fig. S6a) with a radius of gyration of 17.8 ±1.2 nm. An attempt was made to measure the scattering profile of B8K5 by SANS with contrast variation from mixtures of normal and heavy water on the D11 spectrometer at the Institut Max Von Laue_/Paul Langevin in Grenoble (France). The measurement conditions were the same as reported previously for native casein micelles (Holt et al., 2003). The SANS profile of the B8K5 sample in the aqueous buffer is shown in Fig. S6b but in 65% and 100% D_2_O the samples showed signs of instability. In subsequent work designed to enable contrast variation experiments to be performed, the stability of the particles in D_2_O was improved by removing the Mg salt from the buffer formulations and increasing the proportion of the more soluble free caseins to give α = 0.5. Although stable particles were prepared by this means, their size was increased by the addition of D_2_O, as described in the main text.


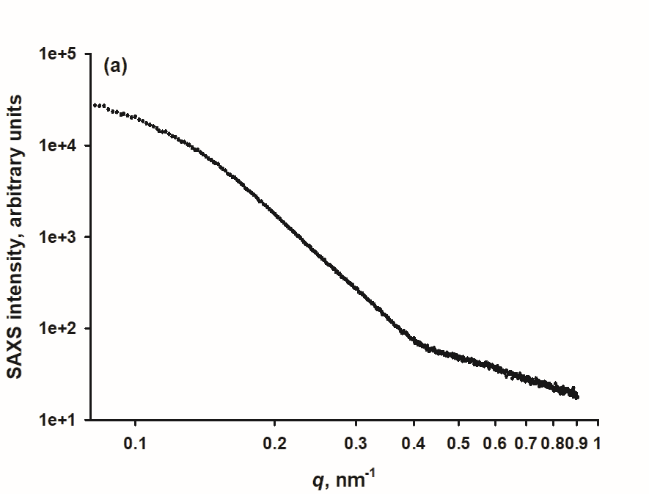

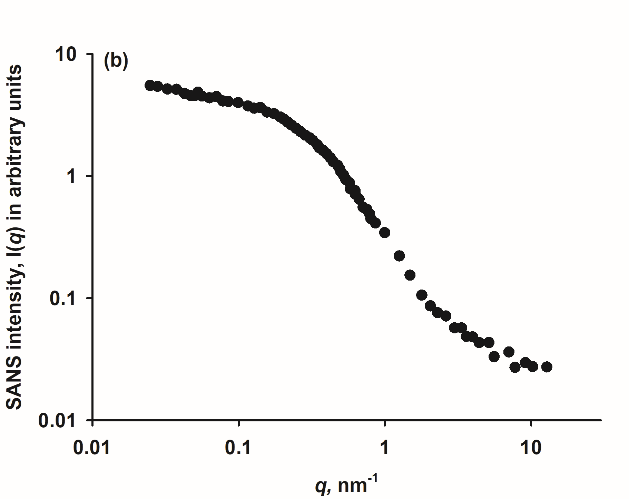


### Figure S6. SAXS and SANS of B8K5 biomimetic casein micelles in the Mg-containing aqueous buffer with α = 1. (a) A SAXS profile recorded with a camera length of 8 m, giving a root mean square Z-average radius of gyration of 17.8 nm, calculated from the Guinier region. (b) The SANS profile in 100% H_2_O. In 65% and 100% D_2_O the samples showed an upturn in scattering intensity at low *q*.

## References

Andrews, A.L., Atkinson, D., Evans, M.T.A., Finer, E.G., Green, J.P., Phillips, M.C., Robertson, R.N., 1979. Conformation and aggregation of bovine beta casein-A 1. Molecular aspects of thermal association. Biopolymers 18, 1105-1121.

Barron, L.D., Blanch, E.W., Hecht, L., 2002. Unfolded proteins studied by raman optical activity. Unfolded Proteins 62, 51-90.

Berry, G.P., Creamer, L.K., 1975. Association of bovine beta-casein- importance of the C-terminal region. Biochemistry 14, 3542-3545.

Bijl, E., Huppertz, T., van Valenberg, H., Holt, C., 2019. A quantitative model of the bovine casein micelle: ion equilibria and calcium phosphate sequestration by individual caseins in bovine milk. European Biophysics Journal 48, 45-59.

Bouchoux, A., Ventureira, J., Gesan-Guiziou, G., Garnier-Lambrouin, F., Qu, P., Pasquier, C., Pezennec, S., Schweins, R., Cabane, B., 2015. Structural heterogeneity of milk casein micelles: A SANS contrast variation study. Soft Matter 11, 389-399.

Bu, H.Y., Sood, S.M., Slattery, C.W., 2004. The effect of C-terminal deletion on the folding and self-association of recombinant non-phosphorylated human beta-casein. Protein Journal 23, 509-517.

Day, L., Raynes, J.K., Leis, A., Liu, L.H., Williams, R.P.W., 2017. Probing the internal and external micelle structures of differently sized casein micelles from individual cows milk by dynamic light and small-angle X-ray scattering. Food Hydrocolloids 69, 150-163.

de Kruif, C.G., 2014. The structure of casein micelles: a review of small-angle scattering data. Journal of Applied Crystallography 47, 1479-1489.

Drake, A.F., Siligardi, G., Gibbons, W.A., 1988. Reassessment of the electronic circular dichroism criteria for random coil conformations of poly(l-lysine) and the implications for protein folding and denaturation studies. Biophysical Chemistry 31, 143-146.

Farrell, H.M., Wickham, E.D., Unruh, J.J., Qi, P.X., Hoagland, P.D., 2001. Secondary structural studies of bovine caseins: temperature dependence of beta-casein structure as analyzed by circular dichroism and FTIR spectroscopy and correlation with micellization. Food Hydrocolloids 15, 341-354.

Greenfield, N.J., 2006. Using circular dichroism spectra to estimate protein secondary structure. Nature Protocols 1, 2876-2890.

Hansen, S., Bauer, R., Lomholt, S.B., Quist, K.B., Pedersen, J.S., Mortensen, K., 1996. Structure of casein micelles studied by small-angle neutron scattering. European Biophysics Journal with Biophysics Letters 24, 143-147.

Holt, C., Davies, D.T., Law, A.J.R., 1986. Effects of colloidal calcium phosphate content and free calcium ion concentration in the milk serum on the dissociation of bovine casein micelles. J. Dairy Res. 53, 557-572.

Holt, C., Wahlgren, N.M., Drakenberg, T., 1996. Ability of a beta-casein phosphopeptide to modulate the precipitation of calcium phosphate by forming amorphous dicalcium phosphate nanoclusters. Biochemical Journal 314, 1035-1039.

Holt, C., Sorensen, E.S., Clegg, R.A., 2009. Role of calcium phosphate nanoclusters in the control of calcification. Febs Journal 276, 2308-2323.

Holt, C., Timmins, P.A., Errington, N., Leaver, J., 1998. A core-shell model of calcium phosphate nanoclusters stabilized by beta-casein phosphopeptides, derived from sedimentation equilibrium and small-angle X-ray and neutron-scattering measurements. European Journal of Biochemistry 252, 73-78.

Holt, C., de Kruif, C.G., Tuinier, R., Timmins, P.A., 2003. Substructure of bovine casein micelles by small-angle X-ray and neutron scattering. Colloids and Surfaces a-Physicochemical and Engineering Aspects 213, 275-284.

Ingham, B., Erlangga, G.D., Smialowska, A., Kirby, N.M., Wang, C., Matia-Merino, L., Haverkamp, R.G., Carr, A.J., 2015. Solving the mystery of the internal structure of casein micelles. Soft Matter 11, 2723-2725.

Jacrot, B., Zaccai, G., 1981. Determination of molecular-weight by neutron-scattering. Biopolymers 20, 2413-2426.

Lazaridis, T., Karplus, M., 1999. Effective energy function for proteins in solution. Proteins 35, 133-152.

Little, E.M., Holt, C., 2004. An equilibrium thermodynamic model of the sequestration of calcium phosphate by casein phosphopeptides. European Biophysics Journal with Biophysics Letters 33, 435-447.

Liu, D.S., Li, J.K., Zhang, J., Liu, X.M., Wang, M., Hemar, Y., Regenstein, J.M., Zhou, P., 2017. Effect of partial acidification on the ultrafiltration and diafiltration of skim milk: Physico-chemical properties of the resulting milk protein concentrates. Journal of Food Engineering 212, 55-64.

Maccallum, P.H., Poet, R., Milner-White, E.J., 1995. Coulombic attractions between partially charged main-chain atoms stabilize the right-handed twist found in most beta-strands. J. Mol. Biol. 248, 374-384.

Mao, A.H., Crick, S.L., Vitalis, A., Chicoine, C.L., Pappu, R.V., 2010. Net charge per residue modulates conformational ensembles of intrinsically disordered proteins. Proc. Natl. Acad. Sci. U. S. A. 107, 8183-8188.

Marchin, S., Putaux, J.-L., Pignon, F., Léonil, J., 2007. Effects of the environmental factors on the casein micelle structure studied by cryo transmission electron microscopy and small-angle x-ray scattering/ultrasmall-angle x-ray scattering. J Chem Phys 126, 045101.

Miles, A.J., Janes, R.W., Wallace, B.A., 2021. Tools and methods for circular dichroism spectroscopy of proteins: a tutorial review. Chemical Society Reviews 50, 8400-8413.

O'Connell, J.E., Grinberg, V.Y., de Kruif, C.G., 2003. Association behavior of beta-casein. J. Colloid Interface Sci. 258, 33-39.

Papiz, M.Z., Bellini, D., Evans, K., Grossmann, J.G., Fordham-Skelton, T., 2019. Light-induced complex formation of bacteriophytochrome RpBphP1 and gene repressor RpPpsR2 probed by SAXS. The FEBS Journal 286, 4261-4277.

Pignon, F., Belina, G., Narayanan, T., Paubel, X., Magnin, A., Gesan-Guiziou, G., 2004. Structure and rheological behavior of casein micelle suspensions during ultrafiltration process. J. Chem. Phys. 121, 8138-8146.

Portnaya, I., Avni, S., Kesselman, E., Boyarski, Y., Sukenik, S., Harries, D., Dan, N., Cogan, U., Danino, D., 2016. Competing processes of micellization and fibrillization in native and reduced casein proteins. Phys. Chem. Chem. Phys. 18, 22516-22525.

Qi, P.X., Wickham, E.D., Farrell, H.M., 2004. Thermal and alkaline denaturation of bovine beta-casein. Protein Journal 23, 389-402.

Qi, P.X., Wickham, E.D., Piotrowski, E.G., Fagerquist, C.K., Farrell, H.M., 2005. Implication of C-terminal deletion on the structure and stability of bovine beta-casein. Protein Journal 24, 431-444.

Shi, Z.S., Chen, K., Liu, Z.G., Kallenbach, N.R., 2006. Conformation of the backbone in unfolded proteins. Chem. Rev. 106, 1877-1897.

Singh, R., Hemar, Y., Gilbert, E.P., Wu, Z., Yang, Z., 2020. Effect of genipin cross-linking on the structural features of skim milk in the presence of ethylenediaminetetraacetic acid (EDTA). Colloids and Surfaces A: Physicochemical and Engineering Aspects 603, 125174.

Smyth, E., Clegg, R.A., Holt, C., 2004. A biological perspective on the structure and function of caseins and casein micelles. International Journal of Dairy Technology 57, 121-126.

Syme, C.D., Blanch, E.W., Holt, C., Jakes, R., Goedert, M., Hecht, L., Barron, L.D., 2002. A Raman optical activity study of rheomorphism in caseins, synucleins and tau - New insight into the structure and behaviour of natively unfolded proteins. European Journal of Biochemistry 269, 148-156.

Tompa, P., 2012. Intrinsically disordered proteins: a 10-year recap. Trends in Biochemical Sciences 37, 509-516.

Woody, R.W., 2009. Circular Dichroism Spectrum of Peptides in the Poly(Pro)II Conformation. Journal of the American Chemical Society 131, 8234-8245.
